# Clinical outcomes after fully visualized transforaminal endoscopic spine surgery in patients with lumbar disc herniation stratified by paraspinal muscle degeneration: A retrospective cohort study

**DOI:** 10.1371/journal.pone.0361478

**Published:** 2026-11-03

**Authors:** Li Wang, Bin Shi, Weiliang Zhang, Xiaoliang Ma, Bin Cao, Hui Xiao, Haigang Zhang

**Affiliations:** 1 Department of Nursing, Xi’an People’s Hospital (Xi’an Fourth Hospital), Xi’an, Shaanxi, China; 2 Department of Pain Medicine, Xi’an People’s Hospital (Xi’an Fourth Hospital), Xi’an, Shaanxi, China; University of California San Diego, UNITED STATES OF AMERICA

## Abstract

**Objective:**

To evaluate pain and functional outcomes after fully visualized transforaminal endoscopic spine surgery in patients with lumbar disc herniation (LDH) stratified by preoperative paraspinal muscle degeneration, and to examine whether degeneration grade was associated with 2-year outcomes.

**Methods:**

This retrospective cohort study included 129 patients with single-level LDH who underwent surgery between December 2021 and December 2023 and had complete preoperative, 1-month, 6-month, and 2-year follow-up data. Two blinded spine surgeons independently graded bilateral multifidus fatty infiltration from 0 to 4 on axial T2-weighted MRI at the mid-disc level; linearly weighted kappa was 0.967(95% CI, 0.937 to 0.989). Longitudinal outcomes were analyzed using covariate-adjusted patient-level random-intercept mixed-effects models with degeneration grade treated as a categorical factor, followed by adjusted 2-year regression and post hoc sensitivity analyses.

**Results:**

Of 136 screened patients, 129 were included after excluding 2 with incomplete records and 5 lost to follow-up. The cohort included 64 men and 65 women, with a mean age of 57.36 ± 11.63 years; grades 0–4 included 13, 25, 33, 32 and 26 patients, respectively. All grades improved substantially. In covariate-adjusted mixed models treating grade as a factor, the grade effect was significant for back pain VAS (P < 0.001), leg pain VAS (P = 0.010), and ODI (P < 0.001). The time-by-grade interaction was nominally significant for leg pain VAS (P = 0.036) but not after false-discovery-rate adjustment (q = 0.053), and not significant for back VAS and ODI. Each one-grade increase was associated with higher 2-year back pain VAS (β = 0.114, 95% CI, 0.001 to 0.227, P = 0.048) and ODI (β = 1.001, 95% CI, 0.168 to 1.833, P = 0.019), but not leg pain VAS.

**Conclusions:**

Fully visualized transforaminal endoscopic spine surgery was associated with substantial pain relief and functional improvement across all multifidus degeneration grades. More severe degeneration showed modest prognostic associations with residual back pain and disability, but the estimated effects were smaller than published minimal clinically important differences. These findings should inform cautious counseling and hypothesis generation rather than be used as a standalone surgical decision rule.

## Introduction

Lumbar disc herniation (LDH) is a common cause of low back pain, radicular leg pain, sensory disturbance, and functional limitation in adults [1,2]. Although many patients improve with conservative treatment, surgical decompression remains an important option for persistent radiculopathy with imaging-confirmed nerve root compression [3,4]. With advances in endoscopic visualization, working-channel systems, and foraminoplasty techniques, full-endoscopic lumbar discectomy has become increasingly used because it aims to preserve posterior soft tissues while achieving targeted neural decompression [5,6].

Clinical recovery after endoscopic surgery is heterogeneous. In addition to herniation characteristics, disease duration, surgical level, and technical factors, the condition of the paraspinal muscles may influence residual pain and functional recovery. In patients with LDH, multifidus degeneration varies with age, sex, and herniation-side characteristics, and MRI-detected paraspinal muscle changes have been linked to local inflammatory processes and back pain [7,8]. Systematic and mechanistic evidence suggests that altered lumbar muscle composition is associated with low back pain and disc degeneration, although causality remains difficult to establish [9,10].

Previous studies have linked paraspinal muscle fatty infiltration with worse patient-reported outcomes after lumbar discectomy [11,12]. Moderate-to-severe multifidus fatty atrophy has been associated with recurrent herniation after microdiscectomy [13], whereas broader evidence links multifidus fatty infiltration to postoperative disability and persistent low back pain after lumbar surgery [14]. In endoscopic cohorts, residual back pain, incomplete clinical improvement, and reoperation remain clinically relevant despite generally favorable outcomes [15,16]. However, the longitudinal pattern of recovery across semiquantitative degeneration grades after fully visualized transforaminal endoscopic surgery has not been fully described. We therefore analyzed pain and functional trajectories in LDH patients stratified by modified Goutallier grade and evaluated the adjusted association between degeneration grade and 2-year outcomes.

## Materials and methods

### Study design and participants

This retrospective cohort study screened 136 patients who underwent single-level fully visualized transforaminal endoscopic spine surgery for LDH from December 2021 to December 2023. Two patients were excluded because of incomplete records and five were lost to follow-up, leaving 129 patients with complete preoperative, 1-month, 6-month, and 2-year VAS/ODI data for analysis (Fig 1). Authorized investigators accessed institutional clinical records for research analysis on 1 May 2026. The analytic dataset was de-identified before analysis.

**Fig 1:**
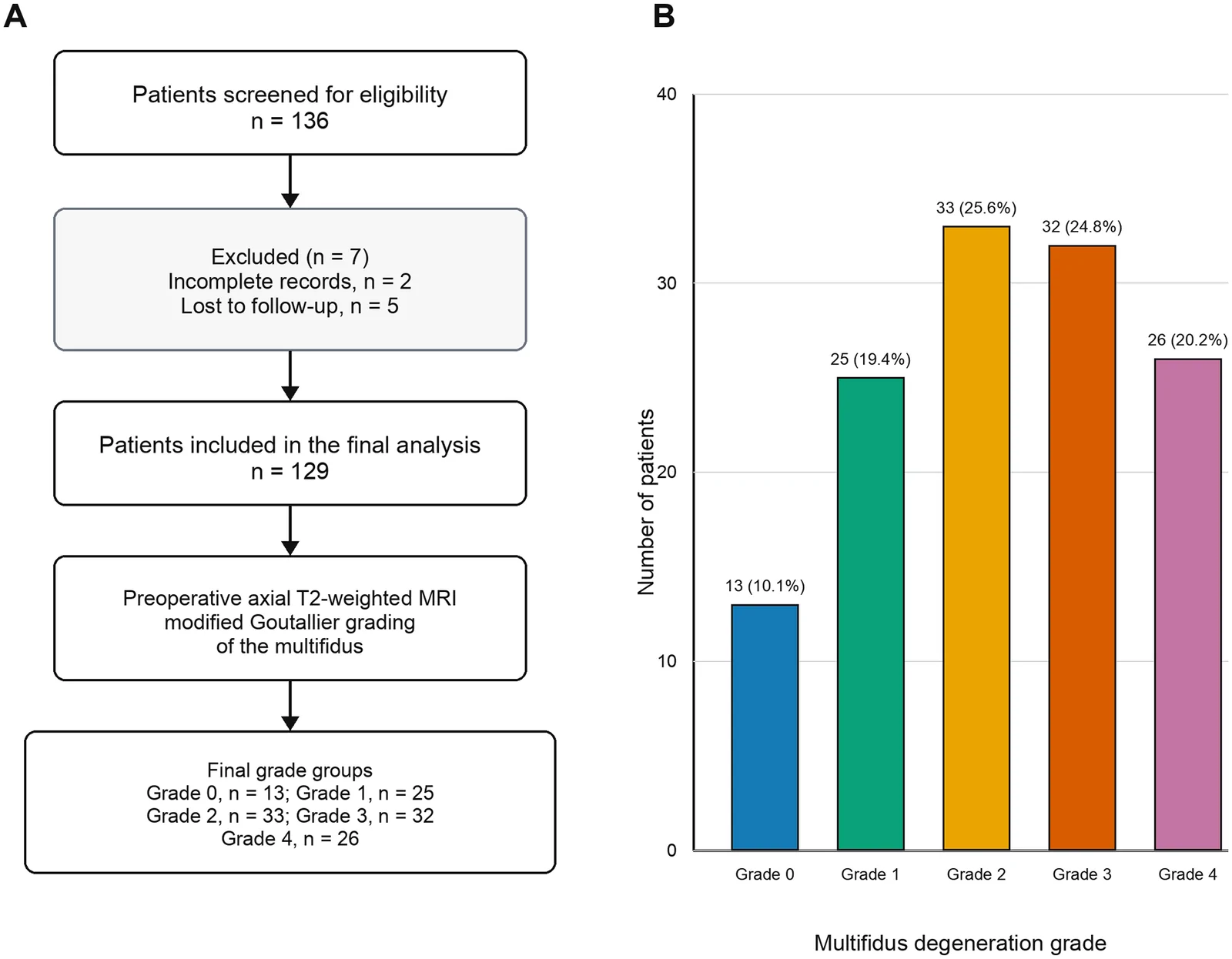
Study flow and distribution of multifidus degeneration grades. (A) Of 136 patients screened for eligibility, 2 were excluded because of incomplete records and 5 were lost to follow-up, leaving 129 patients with complete preoperative, 1-month, 6-month, and 2-year outcome data for the final analysis. (B) Distribution of the modified Goutallier grades in the analyzed cohort: grade 0, 13 patients (10.1%); grade 1, 25 (19.4%); grade 2, 33 (25.6%); grade 3, 32 (24.8%); and grade 4, 26 (20.2%). Grades were assigned from the combined bilateral appearance of the multifidus on preoperative axial T2-weighted MRI at the midpoint of the responsible intervertebral disc. Percentages are based on 129 patients and may not total 100% because of rounding. MRI, magnetic resonance imaging.

Patients were eligible if they had clinical symptoms, physical signs, and MRI findings consistent with LDH; persistent or recurrent symptoms after standardized conservative treatment; single-level disease; preoperative MRI suitable for multifidus assessment; first-time single-level transforaminal full-endoscopic surgery; and complete VAS/ODI follow-up data before surgery and at 1 month, 6 months, and 2 years. Exclusion criteria were spondylolisthesis, severe lumbar spinal stenosis, scoliosis, infection, tumor, or fracture; multilevel disease or concurrent lumbar surgery; previous lumbar surgery; severe organ dysfunction or inability to complete follow-up; and missing key clinical data. Requiring complete follow-up may preferentially exclude patients with complications, persistent dysfunction, or lower follow-up engagement; this potential selection bias was considered when interpreting the observed recovery rates.

### Ethics statement

This retrospective study was reviewed and approved by the Science and Technology Ethics Committee of Xi’an People’s Hospital (Xi’an Fourth Hospital) on April 16, 2025 (approval No. KJLL-Z-K-2025034). The requirement for informed consent was waived by the ethics committee because this study used retrospectively collected clinical data. All data were anonymized before analysis, and the study was conducted in accordance with the principles of the Declaration of Helsinki.

### Paraspinal muscle degeneration grading

Multifidus degeneration was assessed on preoperative axial lumbar T2-weighted MRI at the midpoint of the responsible intervertebral disc. Bilateral multifidus fatty infiltration was evaluated together and assigned one overall modified Goutallier grade: grade 0, no visible fatty infiltration; grade 1, minimal fatty streaks; grade 2, fatty infiltration involving less than 50% of the muscle area; grade 3, approximately 50% fatty infiltration; and grade 4, fatty infiltration involving more than 50% of the muscle area [17,18] (Fig 2). Two spine surgeons with 5–10 years of experience independently graded all scans while blinded to postoperative outcomes. Six disagreements were resolved by joint review with an adjudication panel comprising a spine surgeon and a radiologist, each with more than 10 years of experience. Exact agreement was 95.3% (123/129); linearly weighted kappa was 0.967 (bootstrap 95% CI, 0.937 to 0.989). Quadratically weighted kappa was 0.985 (95% CI, 0.971 to 0.995). Repeat readings were not performed, so intra-rater reliability was not assessed.

**Fig 2:**
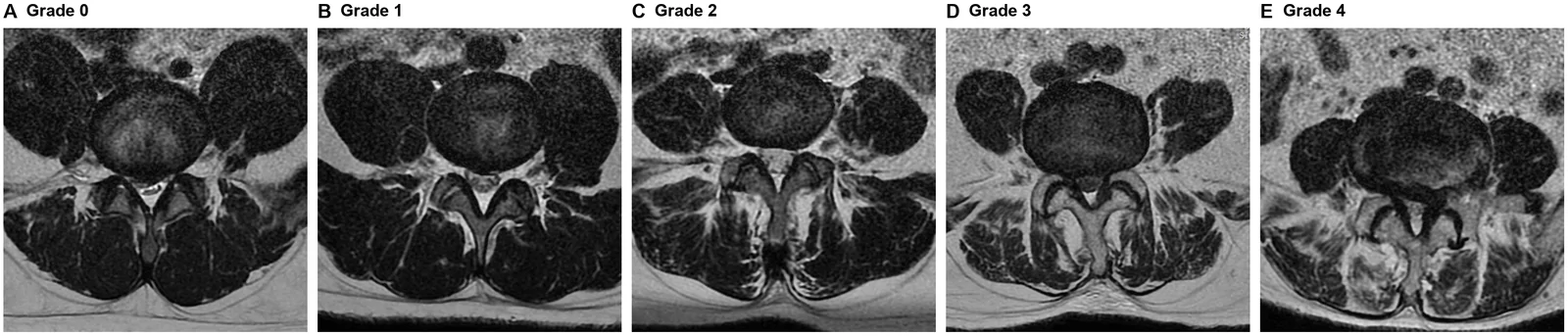
Representative magnetic resonance imaging appearances of multifidus fatty infiltration by modified Goutallier grade. Representative preoperative axial T2-weighted images obtained at the midpoint of the responsible intervertebral disc illustrate the combined bilateral assessment of the multifidus: (A) grade 0, no visible fatty infiltration; (B) grade 1, minimal fatty streaks; (C) grade 2, fatty infiltration involving less than 50% of the muscle area; (D) grade 3, approximately 50% fatty infiltration; and (E) grade 4, fatty infiltration involving more than 50% of the muscle area. One overall grade was assigned from the combined appearance of the right and left multifidus muscles. MRI, magnetic resonance imaging.

### Surgical procedure and postoperative care

All patients underwent single-level fully visualized transforaminal endoscopic spine surgery, performed by the same spine surgeon using a consistent operative and postoperative protocol, following contemporary full-endoscopic principles of targeted decompression under continuous visualization [19,20]. Patients were positioned prone, and the responsible level was localized using C-arm fluoroscopy. The puncture point and angle were selected according to the target level and body habitus. After percutaneous puncture, sequential dilation, and working-channel placement, foraminoplasty, ligamentum flavum management, and removal of the herniated nucleus pulposus were performed under direct endoscopic visualization. Decompression was considered adequate when the affected nerve root was fully released and dural pulsation had recovered. Postoperatively, all patients received the same protocol of bed rest, early ambulation with lumbar bracing, straight-leg-raising exercise, and lumbar extensor training.

### Outcomes

Baseline and perioperative variables included age, sex, BMI, disease duration, herniation type according to the Michigan State University classification, side of herniation, surgical segment, operative time, and estimated blood loss. Clinical outcomes were back pain VAS and leg pain VAS, each scored from 0 (no pain) to 10 (worst imaginable pain) [21], and the validated Chinese-language Oswestry Disability Index version 2.1a (ODI; 0–100, with higher scores indicating greater disability) [22,23]. Outcomes were collected from inpatient and outpatient records before surgery and at 1 month, 6 months, and 2 years; when a scheduled outcome was incompletely documented, trained research staff administered the same questionnaire by telephone. Recurrence and reoperation within 2 years were abstracted where documented. Modified MacNab scores, JOA scores, and complications were not completely recorded and therefore were not included in inferential analyses.

### Statistical analysis

Statistical reanalyses were performed using R version 4.6.0 (R Foundation for Statistical Computing, Vienna, Austria). Continuous variables were summarized as mean ± standard deviation, and categorical variables as counts and percentages. Between-group comparisons used Kruskal-Wallis tests for continuous variables and chi-square tests for categorical variables; Monte Carlo simulation was used when expected cell counts were low. These single-time-point comparisons were descriptive.

Longitudinal changes in back pain VAS, leg pain VAS, and ODI were analyzed using linear mixed-effects models with patient-level random intercepts. Fixed effects included time, degeneration grade as a five-level categorical factor, the time-by-grade interaction, age, sex, BMI, disease duration, and surgical segment; sum-to-zero contrasts were used and fixed effects were evaluated with Wald chi-square tests. Benjamini-Hochberg false-discovery-rate (FDR) q values were calculated across the six exposure-related primary tests (three grade effects and three time-by-grade interactions). Multivariable linear regression quantified the adjusted linear trend per one-grade increase in 2-year outcomes, controlling for the corresponding baseline score, age, sex, BMI, disease duration, and surgical segment. The grade-specific partial R-squared was calculated from the reduction in residual sum of squares after adding grade to the covariate-only model; change in adjusted R-squared was also reported. Post hoc 2-year analyses treating grade as a factor or as grades 0–2 versus 3–4 were exploratory; their nominal P values were not multiplicity-adjusted. Improvement-score models were also post hoc and were moved to the Supporting Information because improvement equals baseline minus the 2-year value and is mathematically coupled to both. Two-sided P < 0.05 was used for nominal tests, with effect estimates and confidence intervals emphasized.

## Results

### Patient characteristics

Of 136 screened patients, 2 were excluded because of incomplete records and 5 were lost to follow-up; 129 were included in the final analysis (Fig 1). The cohort comprised 64 men (49.6%) and 65 women (50.4%), with a mean age of 57.36 ± 11.63 years and a mean BMI of 23.89 ± 2.29 kg/m2. Modified Goutallier grades 0, 1, 2, 3, and 4 included 13 (10.1%), 25 (19.4%), 33 (25.6%), 32 (24.8%), and 26 (20.2%) patients, respectively. Age differed among degeneration grades (P = 0.003), with higher mean age in grades 3 and 4. The remaining measured baseline and perioperative characteristics did not differ significantly (Table 1). During 2-year follow-up, three recurrences were documented (2.3%): one in grade 3 and two in grade 4; one patient underwent reoperation. Complication data were incomplete and were not analyzed.

**Table 1:** Baseline demographic, clinical, imaging, and perioperative characteristics according to multifidus degeneration grade.

Variable	Overall	Grade 0	Grade 1	Grade 2	Grade 3	Grade 4	P value
Age, years	57.36 ± 11.63	54.31 ± 9.06	52.12 ± 9.36	54.91 ± 14.97	62.62 ± 9.81	60.54 ± 8.77	P = 0.003
Sex, n (%): Male	64 (49.6%)	7 (53.8%)	9 (36.0%)	20 (60.6%)	17 (53.1%)	11 (42.3%)	P = 0.373
Female	65 (50.4%)	6 (46.2%)	16 (64.0%)	13 (39.4%)	15 (46.9%)	15 (57.7%)	
BMI, kg/m2	23.89 ± 2.29	22.92 ± 2.41	23.73 ± 2.11	24.14 ± 2.22	23.87 ± 2.51	24.21 ± 2.25	P = 0.572
Disease duration, months	15.17 ± 12.36	9.85 ± 8.60	16.84 ± 14.36	16.91 ± 13.50	11.84 ± 9.62	18.12 ± 12.50	P = 0.162
Herniation type, n (%): MSU 1	19 (14.7%)	1 (7.7%)	2 (8.0%)	6 (18.2%)	8 (25.0%)	2 (7.7%)	P = 0.277
MSU 2	73 (56.6%)	8 (61.5%)	17 (68.0%)	18 (54.5%)	18 (56.2%)	12 (46.2%)	
MSU 3	37 (28.7%)	4 (30.8%)	6 (24.0%)	9 (27.3%)	6 (18.8%)	12 (46.2%)	
Side of herniation, n (%): Left	58 (45.0%)	8 (61.5%)	8 (32.0%)	15 (45.5%)	14 (43.8%)	13 (50.0%)	P = 0.786
Right	55 (42.6%)	4 (30.8%)	14 (56.0%)	15 (45.5%)	13 (40.6%)	9 (34.6%)	
Bilateral	16 (12.4%)	1 (7.7%)	3 (12.0%)	3 (9.1%)	5 (15.6%)	4 (15.4%)	
Surgical segment, n (%): Other	7 (5.4%)	0 (0.0%)	2 (8.0%)	2 (6.1%)	2 (6.2%)	1 (3.8%)	P = 0.973
L4/5	65 (50.4%)	8 (61.5%)	11 (44.0%)	15 (45.5%)	17 (53.1%)	14 (53.8%)	
L5/S1	57 (44.2%)	5 (38.5%)	12 (48.0%)	16 (48.5%)	13 (40.6%)	11 (42.3%)	
Operative time, min	70.96 ± 14.77	68.62 ± 15.25	70.80 ± 17.20	71.79 ± 13.40	72.88 ± 14.91	68.88 ± 14.22	P = 0.849
Estimated blood loss, mL	33.59 ± 11.16	29.92 ± 9.30	37.32 ± 10.54	31.88 ± 9.54	35.94 ± 12.72	31.12 ± 11.60	P = 0.073

Note: Data are presented as mean ± SD or n (%). Between-group comparisons were performed using Kruskal-Wallis tests for continuous variables and chi-square tests or Monte Carlo simulation for categorical variables, as appropriate.

### Longitudinal changes in clinical outcomes

Back pain VAS decreased from 5.05 ± 1.40 preoperatively to 0.74 ± 0.73 at 2 years. Leg pain VAS decreased from 6.47 ± 1.46 to 0.59 ± 0.77, and ODI decreased from 62.42 ± 11.58 to 12.27 ± 5.57. All degeneration grades showed marked improvement in all three outcomes. Descriptive between-grade comparisons showed differences in back pain VAS at 1 month and 6 months, leg pain VAS at 6 months, and ODI at 6 months and 2 years (Table 2 and Fig 3).

**Table 2:** Clinical outcomes over time according to multifidus degeneration grade.

Outcome	Time point	Grade 0 (n = 13)	Grade 1 (n = 25)	Grade 2 (n = 33)	Grade 3 (n = 32)	Grade 4 (n = 26)	Between-group P value
Back pain VAS	preoperative	4.38 ± 1.04	4.68 ± 1.38	5.09 ± 1.55	5.19 ± 1.42	5.50 ± 1.24	P = 0.086
Back pain VAS	1 month	1.38 ± 0.77	0.64 ± 0.76	1.06 ± 0.93	1.56 ± 1.19	1.69 ± 1.01	P = 0.001
Back pain VAS	6 months	0.69 ± 0.75	0.60 ± 0.58	0.58 ± 0.66	1.44 ± 0.91	1.12 ± 0.82	P < 0.001
Back pain VAS	2 years	0.54 ± 0.52	0.80 ± 0.71	0.55 ± 0.62	0.69 ± 0.74	1.08 ± 0.89	P = 0.126
Leg pain VAS	preoperative	6.69 ± 1.25	6.40 ± 1.26	6.39 ± 1.64	6.38 ± 1.50	6.62 ± 1.55	P = 0.925
Leg pain VAS	1 month	1.38 ± 1.89	1.92 ± 1.89	1.79 ± 1.75	2.19 ± 1.26	2.00 ± 1.26	P = 0.383
Leg pain VAS	6 months	0.38 ± 0.87	0.32 ± 0.48	0.27 ± 0.52	1.41 ± 1.24	1.62 ± 1.17	P < 0.001
Leg pain VAS	2 years	0.62 ± 0.87	0.32 ± 0.56	0.45 ± 0.67	0.91 ± 0.89	0.62 ± 0.75	P = 0.071
ODI	preoperative	55.38 ± 13.07	60.40 ± 11.92	63.61 ± 10.87	64.22 ± 9.38	64.15 ± 12.99	P = 0.144
ODI	1 month	24.62 ± 6.99	22.72 ± 4.95	22.27 ± 4.00	27.41 ± 9.36	24.62 ± 6.60	P = 0.173
ODI	6 months	11.62 ± 3.10	13.12 ± 4.18	12.73 ± 4.80	17.44 ± 7.90	16.46 ± 6.29	P < 0.001
ODI	2 years	12.15 ± 5.26	10.00 ± 3.71	10.88 ± 3.98	15.22 ± 6.70	12.65 ± 6.10	P = 0.009

Note: Data are presented as mean ± SD. Between-group P values refer to comparisons among the five degeneration grades at each time point and were considered descriptive. Longitudinal effects were assessed using linear mixed-effects models and are reported in Table 3.

**Fig 3:**
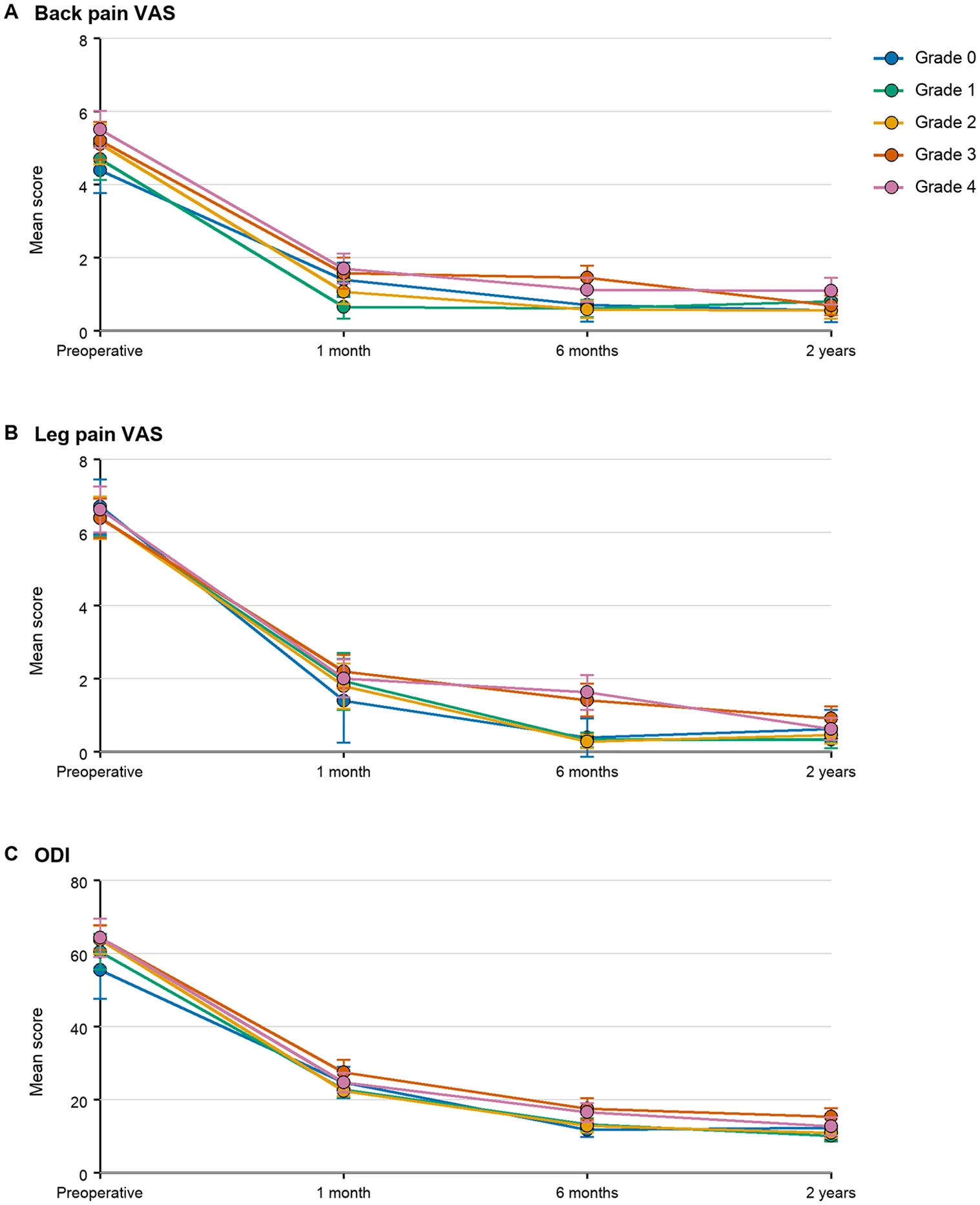
Unadjusted longitudinal clinical outcomes according to multifidus degeneration grade. Mean scores are shown before surgery and at 1 month, 6 months, and 2 years after surgery for (A) back pain visual analogue scale, (B) leg pain visual analogue scale, and (C) Oswestry Disability Index. Points represent observed group means, and error bars represent 95% confidence intervals. Colors identify modified Goutallier grades 0–4 consistently across panels. Group sizes were grade 0, n = 13; grade 1, n = 25; grade 2, n = 33; grade 3, n = 32; and grade 4, n = 26. All 129 patients contributed measurements at each time point. The visual analogue scale ranges from 0 to 10, with higher scores indicating greater pain; the Oswestry Disability Index ranges from 0 to 100, with higher scores indicating greater disability. The plotted values are descriptive and unadjusted; covariate-adjusted longitudinal model results are reported in Table 3. ODI, Oswestry Disability Index; VAS, visual analogue scale.

**Table 3:** Covariate-adjusted linear mixed-effects model results for longitudinal clinical outcomes.

Outcome	Time Wald chi-square (df)	P	Grade Wald chi-square (df)	P/FDR q	Time x grade Wald chi-square (df)	Time x grade Wald chi-square (df)
Back pain VAS	1413.06 (3)	<0.001	37.00 (4)	<0.001 / < 0.001	19.32 (12)	0.081/0.097
Leg pain VAS	1880.29 (3)	<0.001	13.29 (4)	0.010/0.020	22.17 (12)	0.036/0.053
ODI	3064.00 (3)	<0.001	27.91 (4)	<0.001 / < 0.001	12.95 (12)	0.373/0.373

Note: Models included categorical time, degeneration grade as a five-level factor, their interaction, age, sex, BMI, disease duration, and surgical segment as fixed effects, with patient-level random intercepts. Sum-to-zero contrasts and Wald chi-square tests were used. q values are Benjamini-Hochberg FDR-adjusted across the six grade and time-by-grade tests.

### Linear mixed-effects model findings

Covariate-adjusted mixed-effects models treating degeneration grade as a categorical factor showed significant time effects for all outcomes (all P < 0.001). Omnibus grade effects were significant for back pain VAS (Wald χ²(4) = 37.00, P < 0.001, FDR q < 0.001), leg pain VAS (χ²(4) = 13.29, P = 0.010, q = 0.020), and ODI (χ²(4) = 27.91, P < 0.001, q < 0.001). The time-by-grade interaction was nominally significant for leg pain VAS (χ²(12) = 22.17, P = 0.036) but did not remain below 0.05 after FDR adjustment (q = 0.053). Interactions were not significant for back pain VAS (P = 0.081, q = 0.097) or ODI (P = 0.373, q = 0.373) (Table 3). The descriptive trajectories show that leg-pain differences were concentrated at 6 months and had largely converged by 2 years.

### Adjusted associations with 2-year outcomes

In multivariable models adjusted for the corresponding baseline score, age, sex, BMI, disease duration, and surgical segment, each one-grade increase was associated with higher 2-year back pain VAS (β = 0.114; 95% CI, 0.001 to 0.227; P = 0.048) and ODI (β = 1.001; 95% CI, 0.168 to 1.833; P = 0.019), but not leg pain VAS (β = 0.074; 95% CI, −0.038 to 0.186; P = 0.194). Grade contributed partial R-squared values of 0.032, 0.014, and 0.045, respectively; changes in adjusted R-squared were 0.025, 0.006, and 0.038. Even across the full four-grade span, the estimated differences (0.46 points for back pain VAS, 0.30 for leg pain VAS, and 4.00 for ODI) were smaller than published MCIDs of 1.2, 1.6, and 12.8 points [24]. Thus, the statistically detectable associations were modest and should not be interpreted as patient-level treatment effects. Post hoc factor and threshold analyses are presented in S1 Tables, and improvement-score models were moved there because of mathematical coupling (Table 4; Fig 4).

**Table 4:** Adjusted linear trend between multifidus degeneration grade, and 2-year clinical outcomes.

Outcome	Adjusted β per one-grade increase	95% CI	P value	Adjusted R-squared (full)	Adjusted R-squared for grade	Delta adjusted R-squared
2-year back pain VAS	0.114	0.001 to 0.227	P = 0.048	0.009	0.032	0.025
2-year leg pain VAS	0.074	−0.038 to 0.186	P = 0.194	0.021	0.014	0.006
2-year ODI	1.001	0.168 to 1.833	P = 0.019	0.007	0.045	0.038

Note: Beta values represent the adjusted change in the 2-year outcome per one-grade increase. Models adjusted for the corresponding baseline score, age, sex, BMI, disease duration, and surgical segment. Partial R-squared represents the additional variance attributable to grade relative to the covariate-only model; delta adjusted R-squared is the change after adding grade.

**Fig 4:**
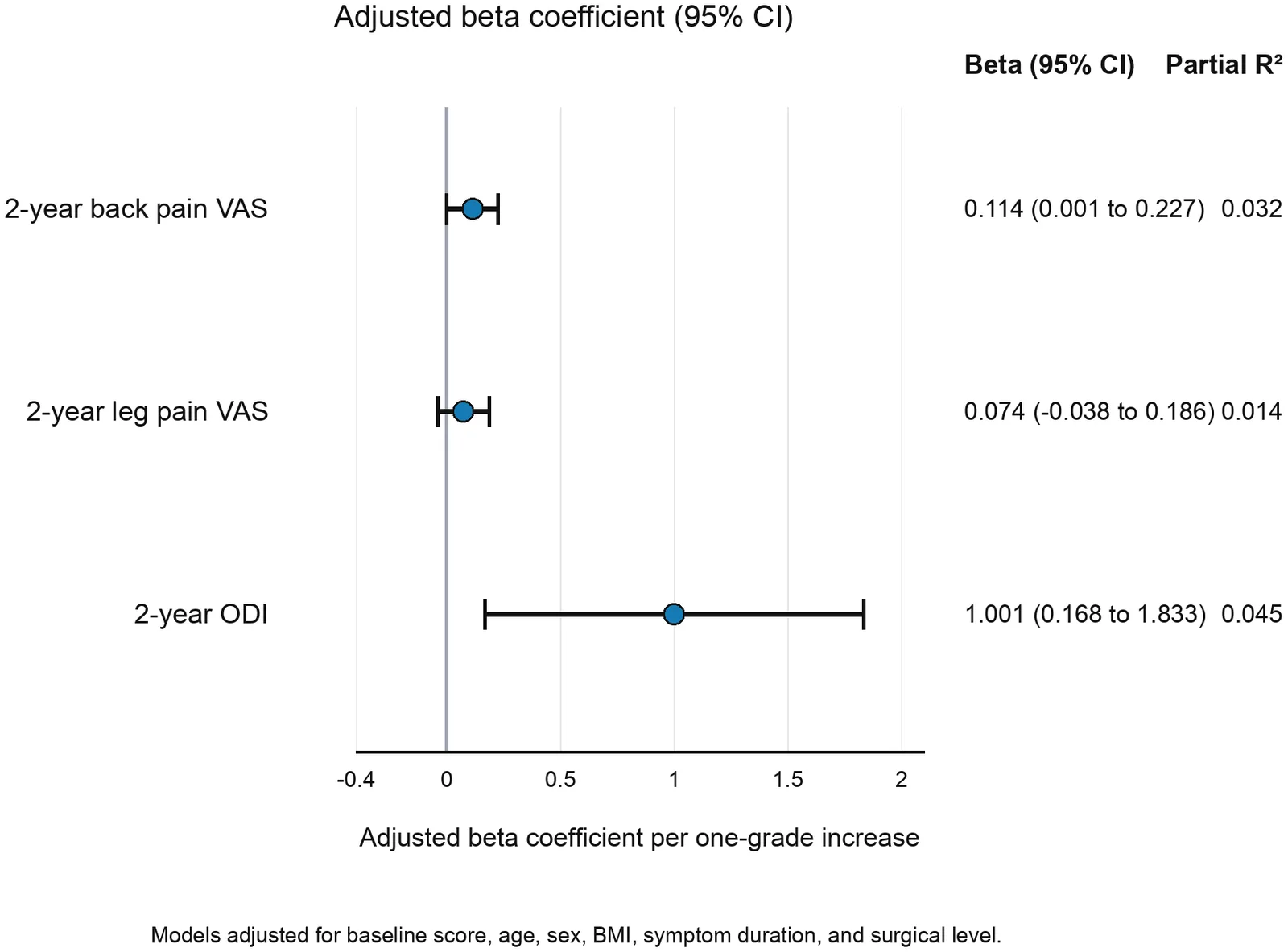
Adjusted associations between multifidus degeneration grade and 2-year clinical outcomes. Points represent adjusted beta coefficients for the difference in each 2-year outcome associated with a one-grade increase in modified Goutallier grade; horizontal lines represent 95% confidence intervals, and the vertical line at zero indicates no association. Positive coefficients indicate higher, and therefore worse, 2-year outcome scores with increasing degeneration grade. Each model included 129 patients and was adjusted for the corresponding baseline outcome score, age, sex, body mass index, symptom duration, and surgical level. Partial R^2^ quantifies the proportion of residual outcome variance explained by degeneration grade after accounting for the prespecified covariates. Back pain and leg pain were assessed on a 0–10 visual analogue scale, and disability was assessed using the 0–100 Oswestry Disability Index. BMI, body mass index; CI, confidence interval; ODI, Oswestry Disability Index; VAS, visual analogue scale.

## Discussion

In this retrospective cohort of 129 patients with single-level LDH, all multifidus degeneration grades showed substantial reductions in back pain, leg pain, and disability after fully visualized transforaminal endoscopic spine surgery. Covariate-adjusted longitudinal models treating grade as a factor found grade-related differences across follow-up, but adjusted 2-year linear-trend estimates were small: approximately 0.11 back-pain VAS points and 1 ODI point per grade, with no significant 2-year leg-pain association. The findings therefore support a modest prognostic association, not a validated risk-stratification tool, surgical contraindication, or standalone decision rule.

The findings are consistent with the direction of recent evidence, but they also refine how that evidence should be applied. A systematic review focused on LDH patients undergoing lumbar discectomy reported that preoperative paraspinal fatty infiltration was associated with recurrence or reoperation, residual low back pain, and poorer functional recovery [11]. A clinical cohort similarly linked paraspinal fatty infiltration with less favorable patient-reported outcomes after lumbar discectomy [12]. Other work has connected multifidus fatty atrophy with recurrence after microdiscectomy and, more broadly, with worse postoperative function after lumbar surgery [13,14]. Our data extend this literature by focusing on a fully visualized transforaminal endoscopic technique, using repeated postoperative time points, and separating three layers of evidence: descriptive between-grade comparisons, longitudinal mixed-effects models, and adjusted 2-year residual outcome models.

The endoscopic context matters. Full-endoscopic lumbar discectomy is not simply a smaller version of open discectomy; it changes the balance between neural decompression, approach-related soft-tissue injury, and postoperative recovery. Meta-analytic and comparative studies suggest that endoscopic approaches can achieve clinical improvements comparable to non-endoscopic discectomy in appropriately selected patients [5,6]. Longer-term comparisons of transforaminal endoscopic discectomy and microdiscectomy also support durable symptom improvement, although recurrence and radiographic changes remain relevant [25]. Recent randomized and comparative studies further emphasize that approach selection, foraminoplasty strategy, and working-channel trajectory can influence technical feasibility and early recovery [20,26]. Prospective evaluation of paraspinal musculature after minimally invasive lumbar decompression and comparisons involving paraspinal muscle-splitting approaches reinforce the importance of approach-related muscle preservation [27,28]. Comparative data across percutaneous transforaminal and biportal endoscopic techniques also show that the term endoscopic surgery includes distinct tissue corridors and learning curves [29]. Because this operation minimizes posterior muscle stripping, the residual differences observed in our study are less likely to reflect surgical muscle injury alone and more likely to reflect the preoperative functional reserve of the paraspinal muscle system.

This interpretation is supported by the biology of paraspinal muscle degeneration. In LDH, multifidus degeneration varies with age, sex, and herniation-side characteristics, indicating that the muscle phenotype is not a random imaging finding [7]. MRI-based studies also suggest links among paraspinal muscle structure, inflammatory processes, and back pain in LDH patients [8]. At a broader level, systematic evidence connects altered lumbar muscle composition with low back pain, while mechanistic studies relate fatty infiltration to disc degeneration and local inflammatory pathways [9,10]. The multifidus, erector spinae, and psoas do not have identical roles: some studies describe muscle-specific degeneration patterns, and sagittal alignment work suggests that paravertebral muscle quality interacts with spinopelvic compensation [30,31]. Therefore, a higher Goutallier grade may represent reduced dynamic stability, diminished extensor endurance, altered load sharing, and impaired postural control rather than a direct failure of endoscopic decompression.

The different behavior of leg pain and ODI is clinically important. Radicular leg pain is primarily driven by nerve root compression and irritation, so adequate decompression can produce substantial relief even when multifidus quality is poor. In the adjusted factor model, the time-by-grade interaction for leg pain was nominally significant (P = 0.036) but the FDR-adjusted q value was 0.053. Descriptive differences were most evident at 6 months, whereas the adjusted 2-year association was not significant (P = 0.194), consistent with early or intermediate separation followed by convergence rather than a persistent long-term grade effect. ODI, by contrast, captures sitting, standing, walking, lifting, personal care, and daily function [22], domains that may depend more on trunk endurance and muscular reserve.

The magnitude of the adjusted associations should be kept in clinical context. A one-grade increase corresponded to approximately 0.114 back-pain VAS points, 0.074 leg-pain points, and 1.001 ODI point at 2 years. These effects, and even their estimated four-grade spans, were smaller than the published MCIDs of 1.2 for back pain, 1.6 for leg pain, and 12.8 for ODI reported in a broader lumbar-surgery population [24]. MCIDs describe within-patient change and are not directly equivalent to between-patient prognostic effects, but they provide useful scale. Grade-specific partial R-squared values of 0.032, 0.014, and 0.045 likewise show that degeneration added only modest explanatory information beyond the prespecified covariates.

The improvement-score models require especially cautious interpretation. Improvement was defined as the preoperative score minus the 2-year score, while baseline score was also included as a predictor. This arithmetic coupling explains why the grade coefficients mirror the 2-year residual-outcome coefficients with opposite signs and why the apparent model fit is much higher. Because these models provide little independent information, they were moved to the Supporting Information and are treated only as post hoc supportive analyses.

Clinically, routine review of paraspinal muscle quality on preoperative MRI may improve counseling and postoperative planning. Patients with advanced degeneration can be told that substantial pain relief remains likely, but that residual disability may be somewhat more persistent and may require longer functional follow-up. This framing is more useful than labeling degeneration as a poor-prognosis finding in isolation. It also aligns with studies showing that recurrence, residual low back pain, and postoperative recovery after PELD are influenced by disc degeneration, Modic changes, annular or endplate factors, and patient activity variables [32–35]. Some investigators have explored adjunctive strategies for discogenic residual back pain after PELD, including sinuvertebral nerve ablation, but such approaches address only selected pain generators [36]. For many patients, the more general target is functional restoration rather than additional tissue ablation.

Rehabilitation should be discussed as a rational implication rather than a proven conclusion of this study. Lumbar kinetic-chain training after percutaneous endoscopic lumbar discectomy has been associated with improved staged recovery, and a recent meta-analysis supports stabilizing and core-strengthening exercise for patients with herniated lumbar disc [37,38]. These findings justify considering structured rehabilitation for patients with grade 3 or 4 degeneration, especially programs emphasizing graded activity, trunk motor control, lumbar extensor endurance, hip-pelvic coordination, and safe return to work or daily activity. Nevertheless, our dataset did not capture rehabilitation intensity, adherence, or exercise progression. Therefore, the present results should not be used to claim that rehabilitation eliminates the effect of paraspinal degeneration. They instead identify a plausible subgroup for future rehabilitation trials.

Several limitations define the boundary of the conclusions. First, the retrospective single-center design limits causal inference. Of 136 screened patients, 2 were excluded for incomplete records and 5 were lost to follow-up; requiring complete follow-up may select against patients with complications, persistent dysfunction, or lower engagement. Baseline data for excluded patients were insufficient for a reliable grade-stratified comparison. Second, the grade 0 included only 13 patients, limiting power and the stability of grade 0-referenced contrasts. Third, age differed across grades, and residual confounding may remain despite covariate adjustment. Fourth, the modified Goutallier classification is semiquantitative. Interobserver reliability was excellent, but repeat readings were not performed and intra-rater reliability was not assessed. Fifth, three recurrences and one reoperation were documented, but the small event count precluded risk modeling; complications, Modified MacNab scores, JOA scores, and rehabilitation adherence were incompletely recorded. Future multicenter prospective studies should use quantitative, muscle-specific MRI, standardized adverse-event and rehabilitation logs, and external validation before degeneration grade is used for individual prediction.

## Conclusions

Fully visualized transforaminal endoscopic spine surgery produced substantial pain relief and functional improvement across all multifidus degeneration grades. Higher degeneration grade had modest associations with residual back pain and ODI, while long-term leg pain converged across grades. The effect estimates were below published MCIDs and explained only a small additional proportion of outcome variance. Multifidus degeneration may therefore be considered a modest prognostic correlate for counseling and research, but it is not a validated predictive marker or a basis for withholding endoscopic decompression.

## Supporting information

S1 Tables Supplementary clinical outcome, sensitivity, and improvement-score analyses. This file contains S1 Table through S4 Table.
(DOCX)

S1 Data Fully anonymized English-language dataset and data dictionary used for the analyses. Exact age has been removed in accordance with the journal’s privacy request.
(XLSX)
