# Supplementary material for: Clinical outcomes after fully visualized transforaminal endoscopic spine surgery in patients with lumbar disc herniation stratified by paraspinal muscle degeneration: A retrospective cohort study
Source: PLoS One. 2026 Nov 3;21(11):e0361478. doi: 10.1371/journal.pone.0361478 (PMC13730341; doi:10.1371/journal.pone.0361478)
Supplement: Supplementary file 1 [file pone.0361478.s001.docx]

**S1-S4 Tables**

*Supplementary clinical outcome, sensitivity, and improvement-score analyses*

**S1 Table. Detailed clinical outcome scores at each time point according to multifidus degeneration grade**

| **Outcome** | **Time point** | **Grade 0** | **Grade 1** | **Grade 2** | **Grade 3** | **Grade 4** | **Between-group P value** |
| --- | --- | --- | --- | --- | --- | --- | --- |
| Back pain VAS | preoperative | 4.38 ± 1.04 | 4.68 ± 1.38 | 5.09 ± 1.55 | 5.19 ± 1.42 | 5.50 ± 1.24 | P = 0.086 |
| Back pain VAS | 1 month | 1.38 ± 0.77 | 0.64 ± 0.76 | 1.06 ± 0.93 | 1.56 ± 1.19 | 1.69 ± 1.01 | P = 0.001 |
| Back pain VAS | 6 months | 0.69 ± 0.75 | 0.60 ± 0.58 | 0.58 ± 0.66 | 1.44 ± 0.91 | 1.12 ± 0.82 | P < 0.001 |
| Back pain VAS | 2 years | 0.54 ± 0.52 | 0.80 ± 0.71 | 0.55 ± 0.62 | 0.69 ± 0.74 | 1.08 ± 0.89 | P = 0.126 |
| Leg pain VAS | preoperative | 6.69 ± 1.25 | 6.40 ± 1.26 | 6.39 ± 1.64 | 6.38 ± 1.50 | 6.62 ± 1.55 | P = 0.925 |
| Leg pain VAS | 1 month | 1.38 ± 1.89 | 1.92 ± 1.89 | 1.79 ± 1.75 | 2.19 ± 1.26 | 2.00 ± 1.26 | P = 0.383 |
| Leg pain VAS | 6 months | 0.38 ± 0.87 | 0.32 ± 0.48 | 0.27 ± 0.52 | 1.41 ± 1.24 | 1.62 ± 1.17 | P < 0.001 |
| Leg pain VAS | 2 years | 0.62 ± 0.87 | 0.32 ± 0.56 | 0.45 ± 0.67 | 0.91 ± 0.89 | 0.62 ± 0.75 | P = 0.071 |
| ODI | preoperative | 55.38 ± 13.07 | 60.40 ± 11.92 | 63.61 ± 10.87 | 64.22 ± 9.38 | 64.15 ± 12.99 | P = 0.144 |
| ODI | 1 month | 24.62 ± 6.99 | 22.72 ± 4.95 | 22.27 ± 4.00 | 27.41 ± 9.36 | 24.62 ± 6.60 | P = 0.173 |
| ODI | 6 months | 11.62 ± 3.10 | 13.12 ± 4.18 | 12.73 ± 4.80 | 17.44 ± 7.90 | 16.46 ± 6.29 | P < 0.001 |
| ODI | 2 years | 12.15 ± 5.26 | 10.00 ± 3.71 | 10.88 ± 3.98 | 15.22 ± 6.70 | 12.65 ± 6.10 | P = 0.009 |

Data are mean +/- SD. This table provides the complete values underlying Table 2 and Fig 3.

**S2 Table. Within-grade changes from baseline to 2 years**

| **Outcome** | **Grade** | **Baseline mean ± SD** | **2-year mean ± SD** | **Mean change** | **95% CI** | **P value** |
| --- | --- | --- | --- | --- | --- | --- |
| Back pain VAS | Grade 0 | 4.38 ± 1.04 | 0.54 ± 0.52 | 3.85 | 3.11 to 4.58 | P < 0.001 |
| Back pain VAS | Grade 1 | 4.68 ± 1.38 | 0.80 ± 0.71 | 3.88 | 3.20 to 4.56 | P < 0.001 |
| Back pain VAS | Grade 2 | 5.09 ± 1.55 | 0.55 ± 0.62 | 4.55 | 3.88 to 5.21 | P < 0.001 |
| Back pain VAS | Grade 3 | 5.19 ± 1.42 | 0.69 ± 0.74 | 4.5 | 3.98 to 5.02 | P < 0.001 |
| Back pain VAS | Grade 4 | 5.50 ± 1.24 | 1.08 ± 0.89 | 4.42 | 3.74 to 5.11 | P < 0.001 |
| Leg pain VAS | Grade 0 | 6.69 ± 1.25 | 0.62 ± 0.87 | 6.08 | 5.21 to 6.95 | P < 0.001 |
| Leg pain VAS | Grade 1 | 6.40 ± 1.26 | 0.32 ± 0.56 | 6.08 | 5.56 to 6.60 | P < 0.001 |
| Leg pain VAS | Grade 2 | 6.39 ± 1.64 | 0.45 ± 0.67 | 5.94 | 5.35 to 6.53 | P < 0.001 |
| Leg pain VAS | Grade 3 | 6.38 ± 1.50 | 0.91 ± 0.89 | 5.47 | 4.82 to 6.12 | P < 0.001 |
| Leg pain VAS | Grade 4 | 6.62 ± 1.55 | 0.62 ± 0.75 | 6.0 | 5.41 to 6.59 | P < 0.001 |
| ODI | Grade 0 | 55.38 ± 13.07 | 12.15 ± 5.26 | 43.23 | 34.49 to 51.97 | P < 0.001 |
| ODI | Grade 1 | 60.40 ± 11.92 | 10.00 ± 3.71 | 50.4 | 44.96 to 55.84 | P < 0.001 |
| ODI | Grade 2 | 63.61 ± 10.87 | 10.88 ± 3.98 | 52.73 | 48.39 to 57.07 | P < 0.001 |
| ODI | Grade 3 | 64.22 ± 9.38 | 15.22 ± 6.70 | 49.0 | 44.64 to 53.36 | P < 0.001 |
| ODI | Grade 4 | 64.15 ± 12.99 | 12.65 ± 6.10 | 51.5 | 45.42 to 57.58 | P < 0.001 |

Mean change was calculated as baseline score minus the 2-year score; positive values indicate improvement. P values are from within-grade paired t tests.

**S3 Table. Post hoc sensitivity analyses for 2-year outcomes**

| **Outcome** | **Model** | **Contrast** | **Adjusted β** | **95% CI** | **P value** | **Adjusted R2** |
| --- | --- | --- | --- | --- | --- | --- |
| 2-year back pain VAS | A: grade as categorical variable | Grade 1 vs Grade 0 | 0.233 | -0.276 to 0.742 | P = 0.367 | 0.024 |
| 2-year back pain VAS | A: grade as categorical variable | Grade 2 vs Grade 0 | 0.005 | -0.486 to 0.497 | P = 0.983 | 0.024 |
| 2-year back pain VAS | A: grade as categorical variable | Grade 3 vs Grade 0 | 0.206 | -0.292 to 0.705 | P = 0.414 | 0.024 |
| 2-year back pain VAS | A: grade as categorical variable | Grade 4 vs Grade 0 | 0.563 | 0.031 to 1.096 | P = 0.038 | 0.024 |
| 2-year back pain VAS | B: Grade 0-2 vs Grade 3-4 | Moderate-to-severe vs mild | 0.287 | 0.005 to 0.569 | P = 0.046 | 0.01 |
| 2-year leg pain VAS | A: grade as categorical variable | Grade 1 vs Grade 0 | -0.285 | -0.810 to 0.241 | P = 0.286 | 0.045 |
| 2-year leg pain VAS | A: grade as categorical variable | Grade 2 vs Grade 0 | -0.145 | -0.646 to 0.356 | P = 0.567 | 0.045 |
| 2-year leg pain VAS | A: grade as categorical variable | Grade 3 vs Grade 0 | 0.276 | -0.229 to 0.780 | P = 0.282 | 0.045 |
| 2-year leg pain VAS | A: grade as categorical variable | Grade 4 vs Grade 0 | -0.018 | -0.548 to 0.513 | P = 0.948 | 0.045 |
| 2-year leg pain VAS | B: Grade 0-2 vs Grade 3-4 | Moderate-to-severe vs mild | 0.306 | 0.024 to 0.589 | P = 0.034 | 0.043 |
| 2-year ODI | A: grade as categorical variable | Grade 1 vs Grade 0 | -2.025 | -5.782 to 1.731 | P = 0.288 | 0.078 |
| 2-year ODI | A: grade as categorical variable | Grade 2 vs Grade 0 | -0.88 | -4.502 to 2.741 | P = 0.631 | 0.078 |
| 2-year ODI | A: grade as categorical variable | Grade 3 vs Grade 0 | 3.944 | 0.274 to 7.615 | P = 0.035 | 0.078 |
| 2-year ODI | A: grade as categorical variable | Grade 4 vs Grade 0 | 1.061 | -2.780 to 4.903 | P = 0.585 | 0.078 |
| 2-year ODI | B: Grade 0-2 vs Grade 3-4 | Moderate-to-severe vs mild | 3.723 | 1.680 to 5.767 | P < 0.001 | 0.062 |

Model A treated grade as a categorical variable with grade 0 as reference. Model B compared grades 0-2 with grades 3-4. Models adjusted for baseline score, age, sex, BMI, disease duration, and surgical segment. These analyses were not prespecified; nominal P values were not adjusted for multiplicity and should be interpreted as exploratory.

**S4 Table. Post hoc improvement-score models**

| **Outcome** | **Adjusted beta per one-grade increase** | **95% CI** | **P value** | **Adjusted R-squared** |
| --- | --- | --- | --- | --- |
| Improvement in back pain VAS | -0.114 | -0.227 to -0.001 | 0.048 | 0.800 |
| Improvement in leg pain VAS | -0.074 | -0.186 to 0.038 | 0.194 | 0.764 |
| Improvement in ODI | -1.001 | -1.833 to -0.168 | 0.019 | 0.826 |

Improvement equals baseline minus the 2-year score. Because baseline was also included as a predictor, these models are mathematically coupled to the primary residual-outcome models and provide little independent information.
