# Supplementary material for: Clinical outcomes after fully visualized transforaminal endoscopic spine surgery in patients with lumbar disc herniation stratified by paraspinal muscle degeneration: A retrospective cohort study
Source: PLoS One. 2026 Nov 3;21(11):e0361478. doi: 10.1371/journal.pone.0361478 (PMC13730341; doi:10.1371/journal.pone.0361478)
Supplement: Supplementary file 2 [file pone.0361478.s004.docx]

Response to the Academic Editor and Reviewers

**PLOS ONE revised submission**

**Manuscript:** *Clinical outcomes after fully visualized transforaminal endoscopic spine surgery in patients with lumbar disc herniation stratified by paraspinal muscle degeneration: a retrospective cohort study*

**Revision date: 19 September 2026**

Dear Academic Editor and Reviewers,

Thank you for your careful and constructive assessment. We have revised the manuscript and resubmission package comprehensively. The major changes include a fully specified MRI grading and adjudication workflow with weighted kappa, covariate-adjusted longitudinal models treating degeneration grade as a categorical factor, false-discovery-rate control, MCID and partial R-squared context, complete cohort-flow reporting, descriptive recurrence and reoperation data, a fully anonymized English-language dataset, and separate Supporting Information tables. Each point is addressed below. Page and line numbers may shift during final typesetting; therefore, stable section, table, figure, and supporting-file locations are provided.

# Summary of major revisions

- MRI assessment: two blinded spine surgeons independently graded bilateral multifidus fatty infiltration on axial T2-weighted MRI at the responsible mid-disc level; exact agreement was 95.3% and linearly weighted kappa was 0.967.
- Statistical analysis: longitudinal mixed models now adjust for age, sex, BMI, symptom duration, and surgical level and treat grade as a five-level factor; FDR q values are reported for the six primary grade-related tests.
- Clinical interpretation: per-grade effects and four-grade spans are compared with published MCIDs, partial R-squared values are reported, and the conclusions are framed as modest prognostic associations rather than a validated risk-stratification tool.
- Cohort and events: 136 patients were screened, 2 were excluded for incomplete records, 5 were lost to follow-up, and 129 were analyzed; three recurrences and one reoperation are reported.
- Transparency and data: the public workbook is English-language, exact age has been removed rather than hidden, and supplementary analyses are supplied separately as Supporting Information.

# Responses to the Journal Requirements

## Journal requirement 1

***Comment:*** *Please ensure that the manuscript meets PLOS ONE style and file-naming requirements.*

**Response:** We revised the manuscript package to follow PLOS ONE conventions. The clean manuscript, marked manuscript, Response to Reviewers, Supporting Information tables, anonymized S1 Data workbook, and individual figure files are provided as separate, clearly named files. The reference list has been standardized to PLOS Vancouver style, including the first six authors followed by et al. when applicable.

**Location:** *Throughout the manuscript and resubmission package.*

## Journal requirement 2

***Comment:*** *Please provide a complete Data Availability Statement.*

**Response:** The Data Availability Statement has been revised to read: ‘All data are in the manuscript and/or supporting information files.’ The de-identified analytic data and data dictionary are supplied as S1 Data.

**Location:** *Data Availability section; S1 Data.*

## Journal requirement 3

***Comment:*** *Remove or anonymize all personal information, including the age column, and do not hide columns containing personal information.*

**Response:** Exact age was removed completely from the released workbook rather than hidden. The workbook contains no names, contact details, dates, hospital identifiers, hidden rows, or hidden columns. A simple sequential record number is used only to distinguish observations. The entirely blank Modified MacNab field was also omitted.

**Location:** *S1 Data workbook, README and Data Dictionary sheets.*

## Journal requirement 4

***Comment:*** *Provide an English-language version of the dataset.*

**Response:** All sheet names, variable names, coding descriptions, README text, and data-dictionary entries are now in English.

**Location:** *S1 Data workbook.*

## Journal requirement 5

***Comment:*** *Remove supplementary tables from the manuscript, upload them as Supporting Information, and list their legends after the references.*

**Response:** Supplementary Tables S1-S4 are supplied in a separate Supporting Information file. The manuscript retains only the legends for S1 Tables and S1 Data after the reference list.

**Location:** *Supporting Information legends after the References; separate Supporting_Information_Tables file.*

## Journal requirement 6

***Comment:*** *Evaluate any reviewer-recommended citations for relevance; citation is not mandatory unless specifically required by the editor.*

**Response:** We evaluated the suggested literature and added the directly relevant lumbar-MRI reliability study by Battaglia et al. and the lumbar-surgery MCID study by Copay et al. We also added the validation study for the simplified Chinese ODI version 2.1a by Liu et al. Other citations were retained only when they directly supported the corresponding statement.

**Location:** *Methods—MRI grading and Outcomes; Discussion—clinical interpretation; References.*

# Reviewer 1

## Comment 1

***Comment:*** *The manuscript contains an unresolved interobserver-reliability placeholder. How many observers were used, and what was the interobserver agreement?*

**Response:** The internal note was removed. Two spine surgeons with 5-10 years of experience independently graded all scans while blinded to postoperative outcomes. Six disagreements were resolved by joint review with an adjudication panel comprising a spine surgeon and a radiologist, each with more than 10 years of experience. Exact agreement was 123/129 (95.3%); linearly weighted kappa was 0.967 (bootstrap 95% CI, 0.937-0.989), and quadratically weighted kappa was 0.985 (95% CI, 0.971-0.995). Repeat readings were not performed; therefore, intra-rater reliability was not assessed and this is acknowledged as a limitation.

**Location:** *Abstract; Materials and methods—Paraspinal muscle degeneration grading; Discussion—Limitations.*

## Comment 2

***Comment:*** *Did any reoperations or recurrences occur during the 2-year follow-up? If unavailable, this should be highlighted as a limitation.*

**Response:** Three recurrences were documented during the 2-year follow-up (2.3%): one in grade 3 and two in grade 4. One patient underwent reoperation. These events are reported descriptively because the small number precluded reliable risk modeling. Complication data were incomplete and were not assumed to be zero.

**Location:** *Results—Patient characteristics; Discussion—Limitations.*

## Comment 3

***Comment:*** *Grade 0 contains only 13 patients, which limits statistical power when it is used as the reference group.*

**Response:** We added group-specific sample sizes to Table 2 and explicitly acknowledged that the small grade 0 subgroup limits statistical power and the stability of grade 0-referenced contrasts.

**Location:** *Table 2; Discussion—Limitations.*

# Reviewer 2

## Comment 1

***Comment:*** *The complete-follow-up requirement may introduce selection bias. Please disclose the excluded subjects and consider whether age and other confounders may systematically favor particular degeneration groups.*

**Response:** We now report the complete cohort flow: 136 patients were screened, 2 were excluded because of incomplete records, 5 were lost to follow-up, and 129 were analyzed. The flowchart displays these numbers. We explain that complete-case inclusion may preferentially exclude patients with complications, persistent dysfunction, or lower follow-up engagement. Baseline information for excluded patients was insufficient for a reliable grade-stratified comparison. The primary mixed models now adjust for age, sex, BMI, symptom duration, and surgical level.

**Location:** *Abstract; Materials and methods—Study design and participants and Statistical analysis; Fig 2; Results—Patient characteristics; Discussion—Limitations.*

## Comment 2

***Comment:*** *Provide clinical context for the small but statistically significant effects by citing MCID values for VAS and ODI.*

**Response:** We added Copay et al. and compare the estimates with MCIDs of 1.2 points for back-pain VAS, 1.6 for leg-pain VAS, and 12.8 for ODI. A one-grade increase corresponded to 0.114, 0.074, and 1.001 points, respectively; even the estimated four-grade spans remained below these MCIDs. We also clarify that MCIDs quantify within-patient change and are not directly equivalent to between-patient prognostic effects.

**Location:** *Results—Adjusted associations with 2-year outcomes; Discussion—Clinical importance; Reference 24.*

## Comment 3

***Comment:*** *Clarify the MRI graders, their qualifications, blinding, and inter- and intra-rater reliability.*

**Response:** The Methods now specify two independent blinded spine surgeons, their experience, the adjudication procedure, the MRI sequence, the evaluated muscle and slice, and both weighted-kappa estimates with 95% confidence intervals. Intra-rater reliability was not assessed because repeat readings were not performed, and this limitation is stated.

**Location:** *Materials and methods—Paraspinal muscle degeneration grading; Discussion—Limitations.*

## Comment 4

***Comment:*** *Report how many patients were excluded because follow-up was incomplete and describe clinically meaningful events, including recurrence, reoperation, complications, MacNab outcomes, and JOA scores.*

**Response:** We added the screened, excluded, lost-to-follow-up, and analyzed counts. Three recurrences and one reoperation are reported. Modified MacNab scores, JOA scores, and complication data were not recorded with sufficient completeness for reliable analysis; therefore, no complication count was inferred and these omissions are clearly listed as limitations.

**Location:** *Fig 2; Results—Patient characteristics and Outcomes; Discussion—Limitations.*

## Minor comment 1

***Comment:*** *The data-access date 05/01/2026 appears to be in the future and may be a typographical error.*

**Response:** The date is correct. To remove numeric ambiguity, it is now written as ‘1 May 2026.’

**Location:** *Materials and methods—Study design and participants.*

## Minor comment 2

***Comment:*** *Explain why the time-by-grade interaction for leg pain was significant while the adjusted 2-year association was not significant.*

**Response:** After covariate adjustment and factor coding, the leg-pain time-by-grade interaction was nominally significant (P = 0.036), but the FDR-adjusted q value was 0.053. Descriptive differences were concentrated at 6 months, whereas the adjusted 2-year association was not significant (P = 0.194). We therefore interpret the pattern as early or intermediate separation followed by convergence rather than a persistent long-term grade effect.

**Location:** *Results—Linear mixed-effects model findings; Discussion—Leg pain and ODI.*

# Reviewer 3 Main Comments

## Main comment 1

***Comment:*** *Remove the internal note and report the number, qualifications, and blinding of the MRI raters, together with inter- and intra-rater reliability.*

**Response:** The internal note was removed. Two blinded spine surgeons with 5-10 years of experience independently graded all MRIs, and six disagreements were adjudicated by a senior spine surgeon and senior radiologist. Exact agreement, linearly weighted kappa, quadratically weighted kappa, and their confidence intervals are reported. Repeat readings were not performed, so intra-rater reliability was unavailable and is acknowledged.

**Location:** *Abstract; Materials and methods—Paraspinal muscle degeneration grading; Discussion—Limitations.*

## Main comment 2

***Comment:*** *The longitudinal mixed models were not adjusted despite significant age differences across grades. Please adjust for age and other prespecified covariates or describe the models as unadjusted.*

**Response:** All primary longitudinal models now adjust for age, sex, BMI, symptom duration, and surgical level in addition to categorical time, categorical grade, and their interaction. The adjusted models are described in Methods and reported in Table 3; interpretation is centered on adjusted estimates.

**Location:** *Materials and methods—Statistical analysis; Results—Linear mixed-effects model findings; Table 3.*

## Main comment 3

***Comment:*** *The linear grade assumption may be inappropriate. Analyze grade as a factor or threshold, state whether sensitivity analyses were prespecified, and address multiple testing.*

**Response:** The primary longitudinal models now treat grade as a five-level categorical factor. Two-year factor models and the grades 0-2 versus 3-4 threshold models are retained as post hoc sensitivity analyses and are explicitly labeled exploratory. Their nominal P values were not multiplicity-adjusted. For the six primary grade and time-by-grade tests, Benjamini-Hochberg FDR q values are reported.

**Location:** *Materials and methods—Statistical analysis; Results—Linear mixed-effects model findings; Table 3; S3 Table.*

## Main comment 4

***Comment:*** *Report the partial R-squared or change in R-squared for degeneration grade, add MCID context, and avoid calling grade a risk-stratification marker without predictive-performance evidence.*

**Response:** Table 4 now reports partial R-squared values for grade of 0.032, 0.014, and 0.045 and changes in adjusted R-squared of 0.025, 0.006, and 0.038 for back pain, leg pain, and ODI, respectively. MCID comparisons were added. ‘Risk-stratification marker’ was replaced by ‘modest prognostic association/correlate,’ and we explicitly state that predictive performance, calibration, and external validation were not evaluated.

**Location:** *Results—Adjusted associations with 2-year outcomes; Table 4; Discussion; Conclusions.*

## Main comment 5

***Comment:*** *Report screened, excluded, and lost-to-follow-up numbers and acknowledge incomplete recurrence, reoperation, complication, MacNab, and JOA information.*

**Response:** The flowchart now reports 136 screened, 2 excluded for incomplete records, 5 lost to follow-up, and 129 analyzed. Three recurrences and one reoperation are described. Complications, Modified MacNab scores, JOA scores, and rehabilitation adherence were incompletely recorded and are explicitly identified as limitations; the conclusions were correspondingly softened.

**Location:** *Fig 2; Results—Patient characteristics; Discussion—Limitations and Conclusions.*

# Reviewer 3 Minor Comments

## Minor comment 1

***Comment:*** *Use an unambiguous data-access date and correct the missing space after the date.*

**Response:** The text now reads ‘1 May 2026,’ with an unambiguous date format and correct spacing.

**Location:** *Materials and methods—Study design and participants.*

## Minor comment 2

***Comment:*** *Specify the MRI sequence, the muscle assessed, the exact slice, whether one or both sides were graded, and how bilateral findings were combined.*

**Response:** We now specify axial T2-weighted MRI, the multifidus, the midpoint of the responsible intervertebral disc, bilateral assessment, and assignment of one overall grade from the combined bilateral appearance.

**Location:** *Materials and methods—Paraspinal muscle degeneration grading.*

## Minor comment 3

***Comment:*** *In addition to the original Goutallier article, cite a lumbar-MRI adaptation or validation and report reliability.*

**Response:** We added Battaglia et al., which evaluates the reliability of the Goutallier classification for lumbar multifidus fatty degeneration on MRI, and we report our interobserver weighted-kappa estimates.

**Location:** *Materials and methods—Paraspinal muscle degeneration grading; Reference 18.*

## Minor comment 4

***Comment:*** *Because improvement-score analyses are mathematically coupled to baseline-adjusted 2-year models, consider moving them to Supporting Information.*

**Response:** The improvement-score models were moved to S4 Table and are labeled post hoc supportive analyses. The manuscript explains the arithmetic coupling and bases primary inference on the adjusted 2-year residual-outcome models.

**Location:** *Materials and methods—Statistical analysis; Discussion; S4 Table.*

## Minor comment 5

***Comment:*** *Add grade-specific group sizes to Table 2 or the Fig 3 legend.*

**Response:** The grade-specific sample sizes (13, 25, 33, 32, and 26 for grades 0-4) were added to the Table 2 column headings.

**Location:** *Table 2.*

## Minor comment 6

***Comment:*** *Define the VAS range and ODI version/language, describe follow-up collection, and report the number of operating surgeons and consistency of surgical/postoperative protocols.*

**Response:** Back- and leg-pain VAS are now defined from 0 to 10. ODI is specified as the validated simplified Chinese version 2.1a scored from 0 to 100. Outcomes were obtained from inpatient and outpatient records; when a scheduled outcome was incompletely documented, trained research staff administered the same questionnaire by telephone. All procedures were performed by the same spine surgeon using consistent operative and postoperative protocols.

**Location:** *Materials and methods—Surgical procedure and postoperative care; Outcomes.*

# Closing

We appreciate the editor’s and reviewers’ detailed guidance. The revision is substantially more transparent regarding selection, measurement reliability, confounding, multiplicity, effect magnitude, missing safety information, data sharing, and the limits of prognostic interpretation. We hope that the revised manuscript now meets PLOS ONE’s publication criteria.

Sincerely,

Dr. Zhang, on behalf of all authors
