# Supplementary material for: Clinical outcomes after fully visualized transforaminal endoscopic spine surgery in patients with lumbar disc herniation stratified by paraspinal muscle degeneration: A retrospective cohort study
Source: PLoS One. 2026 Nov 3;21(11):e0361478. doi: 10.1371/journal.pone.0361478 (PMC13730341; doi:10.1371/journal.pone.0361478)
Supplement: Peer review history

# Peer review: Decision Letter 0

**DOI:** 10.1371/journal.pone.0361478.r001

**Authors:** Rajakumar Anbazhagan

13 Aug 2026

Dear Dr. Zhang,

Thank you for submitting your manuscript to PLOS ONE. After careful consideration, we feel that it has merit but does not fully meet PLOS ONE’s publication criteria as it currently stands. Therefore, we invite you to submit a revised version of the manuscript that addresses the points raised during the review process.

Please submit your revised manuscript by Oct 12 2026 11:59PM. If you will need more time than this to complete your revisions, please reply to this message or contact the journal office at plosone@plos.org. When you're ready to submit your revision, log on to https://www.editorialmanager.com/pone/ and select the 'Submissions Needing Revision' folder to locate your manuscript file.

- A letter that responds to each point raised by the academic editor and reviewer(s). You should upload this letter as a separate file labeled 'Response to Reviewers'.
- A marked-up copy of your manuscript that highlights changes made to the original version. You should upload this as a separate file labeled 'Revised Manuscript with Track Changes'.
- An unmarked version of your revised paper without tracked changes. You should upload this as a separate file labeled 'Manuscript'.

If you would like to make changes to your financial disclosure, please include your updated statement in your cover letter. Guidelines for resubmitting your figure files are available below the reviewer comments at the end of this letter.

If applicable, we recommend that you deposit your laboratory protocols in protocols.io to enhance the reproducibility of your results. Protocols.io assigns your protocol its own identifier (DOI) so that it can be cited independently in the future. For instructions see: https://journals.plos.org/plosone/s/submission-guidelines#loc-laboratory-protocols. Additionally, PLOS ONE offers an option for publishing peer-reviewed Lab Protocol articles, which describe protocols hosted on protocols.io. Read more information on sharing protocols at https://plos.org/protocols?utm_medium=editorial-email&utm_source=authorletters&utm_campaign=protocols.

As the corresponding author, your ORCID iD is verified in the submission system and will appear in the published article. PLOS supports the use of ORCID, and we encourage all coauthors to register for an ORCID iD and use it as well. Please encourage your coauthors to verify their ORCID iD within the submission system before final acceptance, as unverified ORCID iDs will not appear in the published article. *Only*  the individual author can complete the verification step; PLOS staff *cannot*  verify ORCID iDs on behalf of authors.

We look forward to receiving your revised manuscript.

Kind regards,

Academic Editor

PLOS One

**Journal Requirements:**

When submitting your revision, we need you to address these additional requirements.

1. Please ensure that your manuscript meets PLOS ONE's style requirements, including those for file naming. The PLOS ONE style templates can be found at

https://journals.plos.org/plosone/s/file?id=wjVg/PLOSOne_formatting_sample_main_body.pdf and

https://journals.plos.org/plosone/s/file?id=ba62/PLOSOne_formatting_sample_title_authors_affiliations.pdf

2. Please provide a complete Data Availability Statement in the submission form, ensuring you include all necessary access information or a reason for why you are unable to make your data freely accessible. If your research concerns only data provided within your submission, please write "All data are in the manuscript and/or supporting information files" as your Data Availability Statement.

3. We note that there is identifying data in the Supporting Information file < Data.xlsx >. Due to the inclusion of these potentially identifying data, we have removed this file from your file inventory. Prior to sharing human research participant data, authors should consult with an ethics committee to ensure data are shared in accordance with participant consent and all applicable local laws.

Data sharing should never compromise participant privacy. It is therefore not appropriate to publicly share personally identifiable data on human research participants. The following are examples of data that should not be shared:

-Name, initials, physical address

-Ages more specific than whole numbers

-Internet protocol (IP) address

-Specific dates (birth dates, death dates, examination dates, etc.)

-Contact information such as phone number or email address

-Location data

-ID numbers that seem specific (long numbers, include initials, titled “Hospital ID”) rather than random (small numbers in numerical order)

Data that are not directly identifying may also be inappropriate to share, as in combination they can become identifying. For example, data collected from a small group of participants, vulnerable populations, or private groups should not be shared if they involve indirect identifiers (such as sex, ethnicity, location, etc.) that may risk the identification of study participants.

Additional guidance on preparing raw data for publication can be found in our Data Policy (https://journals.plos.org/plosone/s/data-availability#loc-human-research-participant-data-and-other-sensitive-data) and in the following article: http://www.bmj.com/content/340/bmj.c181.long.

Please remove or anonymize all personal information (kindly remove the age data in column D), ensure that the data shared are in accordance with participant consent, and re-upload a fully anonymized data set. Please note that spreadsheet columns with personal information must be removed and not hidden as all hidden columns will appear in the published file.

4. Thank you for providing your underlying data as Supporting Information.

We note that the data set contains text or data that is not in English. Please note that PLOS is an English-language publisher, so we require data sets to be provided in English as well. Please upload an English-language version of your data set.

This will also allow us to determine if your data follows PLOS standards per our Data Availability policy here: https://journals.plos.org/plosone/s/data-availability

5. We notice that your supplementary tables are included in the manuscript file. Please remove them and upload them with the file type 'Supporting Information'. Please ensure that each Supporting Information file has a legend listed in the manuscript after the references list.

6. If the reviewer comments include a recommendation to cite specific previously published works, please review and evaluate these publications to determine whether they are relevant and should be cited. There is no requirement to cite these works unless the editor has indicated otherwise.

[Note: HTML markup is below. Please do not edit.]

Reviewers' comments:

Reviewer's Responses to Questions

**Comments to the Author**

1. Is the manuscript technically sound, and do the data support the conclusions?

Reviewer #1: Yes

Reviewer #2: Yes

Reviewer #3: Partly

**********

2. Has the statistical analysis been performed appropriately and rigorously?

Reviewer #1: Yes

Reviewer #2: Yes

Reviewer #3: No

**********

3. Have the authors made all data underlying the findings in their manuscript fully available?

The PLOS Data policy

Reviewer #1: Yes

Reviewer #2: Yes

Reviewer #3: Yes

**********

4. Is the manuscript presented in an intelligible fashion and written in standard English?

Reviewer #1: Yes

Reviewer #2: Yes

Reviewer #3: Yes

**********

Reviewer #1: Interobserver reliability placeholder (Lines 90–91): Manuscript contains an unresolved author note: "If two independent observers were used, interobserver reliability should be added before submission." How many observers were used? If 2 reviewers assessed the image, report an inter-observer agreement metric (Cohen's)

Did any reoperations or recurrences occur during the 2-year follow up? That is not stated. If not available, I think this should be highlighted as a limitation.

Grade 0 has only 13 patients which is limiting in statistical power. While using Grade 0 as a baseline and comparing against, this sample size should be acknowledged as limiting.

The figures and tables are sound and differentiate well between primary analyses and supportive analyses.

Reviewer #2: Recommendation: Minor Revision

This retrospective cohort study examines whether preoperative paraspinal muscle degeneration affects surgical outcomes in 129 patients undergoing endoscopic lumbar discectomy. The authors tracked pain and disability measures over two years and found that while all patients improved substantially, those with more advanced muscle degeneration had slightly higher residual disability. The paper is well-structured, the research question is clinically relevant, and the dataset appears carefully curated. However, there are several issues that should be addressed to improve methodological transparency and strengthen the interpretation of the results.

Comments

1. The retrospective design of the study might come with the selection bias, so every aspect of the excluded subjects needs to be disclosed in detail. Requiring complete follow-up data at four time points over two years will systematically exclude patients with complications, persistent dysfunction, or loss to follow-up. That's not inherently disqualifying for a retrospective study, but it likely inflates apparent success rates and may bias the comparison across degeneration grades. Were patients in grade 0 groups perhaps more engaged, more compliant with rehabilitation, or differently distributed by age or comorbidity? The authors mention age differed across grades but don't explore whether confounding might systematically favor certain groups.

2. The paper does not provide the clinical context for effect sizes. A 0.11-point change on a 10-point pain scale is statistically significant but of unknown clinical importance. The paper doesn't reference the minimal clinically important difference (MCID) for VAS or ODI in this population. The authors should reference published MCID values for VAS and ODI in this population, and interpret the effect sizes in that context.

3. The paper states that "if two independent observers were used, interobserver reliability should be added before submission" (line 90-91). That seems like a placeholder comment or a comment from previous revisions. Did the authors actually assess interobserver reliability? The authors should expand the methods section to explicitly state whether interobserver reliability was assessed for MRI grading, who performed the grading, and whether the rater was blinded to outcomes. If not assessed, acknowledge this as a limitation.

4. The authors note that Modified MacNab scores "were not completely recorded" and were therefore excluded. They also acknowledge missing data on complications, recurrence, reoperation, and rehabilitation adherence. Since reoperation and recurrence are clinically meaningful events, the authors should mention that how many patients were excluded because follow-up was incomplete? How many had complications not captured in VAS/ODI scores?

Minor technical comments

• Line 16: The data access date (05/01/2026) is in the future, presumably this is a typo for 2024 or 2025?

• The time-by-grade interaction was significant for leg pain (P = 0.004) in the mixed model, yet the adjusted 2-year association was not significant (P = 0.194). This inconsistency isn't explained. Did early recovery differ by grade but then converge or is this just noise? A brief explanation would help readers understand what actually happened.

Reviewer #3: This single-center retrospective study included 129 patients with single-level LDH. Preoperative paraspinal muscle degeneration was graded from 0 to 4 using a modified Goutallier scale, and back-pain VAS, leg-pain VAS and ODI were assessed over two years. The analyses included descriptive comparisons, random-intercept mixed-effects models and adjusted 2-year regression, with categorical and threshold sensitivity analyses in S3 Table. The clear separation of these analyses and the generally cautious interpretation are important strengths.

Main comments

1. The Methods contain what appears to be an internal note (pg.4 ln.90-91): “If two independent observers were used, interobserver reliability should be added before submission.” This should be removed. Because the modified Goutallier grade is the main exposure, please state how many people graded the MRIs, their qualifications, and whether inter- and intra-rater reliability were assessed, preferably using weighted kappa. If only one rater was used, please state this and acknowledge the possibility of grading error or misclassification.

2. Age differed across degeneration grades (P = 0.003), with patients in grades 3 and 4 being older than those in grade 1. However, the longitudinal mixed models included only time, grade and their interaction. The reported grade associations for back pain (P < 0.001), leg pain (P = 0.002) and ODI (P < 0.001) may therefore be confounded by age. Please adjust these models for age and the other prespecified covariates, or clearly describe them as unadjusted and focus interpretation on the adjusted 2-year models, where back pain was borderline (P = 0.048), ODI remained significant (P = 0.019), and leg pain was not significant.

3. S3 Table already examines grade as a categorical variable and compares grades 0–2 with grades 3–4 for the 2-year outcomes. Among the categorical comparisons, only grade 4 versus grade 0 was significant for back pain, while grade 3 versus grade 0 was significant for ODI; none was significant for leg pain. The grades 0–2 versus grades 3–4 comparison was significant for all three outcomes. These mixed patterns suggest possible non-linearity or a threshold effect rather than a consistent per-grade trend. However, the primary longitudinal model still treats grade as a single linear term. Please analyze grade as a factor or as the 0–2 versus 3–4 grouping in the longitudinal model, or justify the linear assumption. Please also state whether the S3 analyses were prespecified and how multiple testing across these models was handled.

4. The adjusted R² values (0.009 for back-pain VAS and 0.007 for ODI) describe the full models, not the contribution of degeneration grade alone. Please report the partial R² for grade, or the change in R² after adding grade to the model. The estimated effects per grade—approximately 0.11 VAS points and 1 ODI point—are modest compared with commonly used MCIDs of 1.2 and 12.8 points, respectively (Copay et al., 2008; doi:10.1016/j.spinee.2007.11.006). Although these MCIDs were derived from a broader lumbar-surgery population and are not directly equivalent to prognostic effects, they provide useful clinical context. Finally, describing grade as a “risk-stratification marker” is too strong without evidence of predictive performance, calibration or external validation. Please provide such evidence or describe it more cautiously as a modest prognostic association..

5. The study included only patients with complete 2-year follow-up, which may introduce selection bias. Although the manuscript acknowledges this limitation, Fig 2 does not report how many patients were screened, excluded or lost to follow-up. Please provide these numbers and reasons, together with degeneration grades or baseline characteristics where available. Recurrence, reoperation, complications, MacNab outcomes and JOA scores were also incompletely recorded. This limits conclusions about whether muscle degeneration should influence surgical decision-making, particularly regarding recurrence and safety.

Minor comments

1. Text (Abstract and Methods): “…accessed for research analysis on 05/01/2026.Paraspinal muscle degeneration…” — a space is missing after the date, and the numeric format is ambiguous; please use an unambiguous date (ex. 1 May 2026).

2. The MRI grading method needs more detail. Please specify the MRI sequence, the muscle or muscles assessed, the exact slice used at the responsible surgical level, and whether grading was performed on one or both sides and how bilateral findings were combined.

3. Ref 17 describes the original rotator-cuff CT classification; please also cite the lumbar-MRI adaptation or validation supporting the method used here. Because degeneration grade is the main exposure, please report the number of raters and the inter- and intra-rater reliability where available.

4. The improvement-score analyses in Table 4 are algebraically linked to the 2-year outcome models because improvement equals baseline minus the 2-year score, while baseline is also included as a predictor. This explains why the grade coefficients have the same magnitude with opposite signs and why the R² values are much higher. The authors appropriately acknowledge this limitation. Because these models provide little independent information, consider moving them to the Supporting Information.

5. Please add the group sizes to Table 2 or the Fig 3 legend (like grades 0-4: n = 13, 25, 33, 32 and 26). This would help readers interpret the trajectories, particularly for the smaller grade-0 group.

6. Please define the VAS range and ODI version/language, and describe how follow-up outcomes were collected. It would also be helpful to report the number of operating surgeons and whether surgical and postoperative protocols were consistent across the cohort.

**********

what does this mean?). If published, this will include your full peer review and any attached files.

If you choose “no”, your identity will remain anonymous but your review may still be made public.

**Do you want your identity to be public for this peer review?**  For information about this choice, including consent withdrawal, please see our Privacy Policy

Reviewer #1: **Yes:**  PAVITHRA KRISHNASWAMI

Reviewer #2: **Yes:**  Sonal Mahajan

Reviewer #3: No

**********

[NOTE: If reviewer comments were submitted as an attachment file, they will be attached to this email and accessible via the submission site. Please log into your account, locate the manuscript record, and check for the action link "View Attachments". If this link does not appear, there are no attachment files.]

To ensure your figures meet our technical requirements, please review our figure guidelines: https://journals.plos.org/plosone/s/figures

You may also use PLOS’s free figure tool, NAAS, to help you prepare publication quality figures: https://journals.plos.org/plosone/s/figures#loc-tools-for-figure-preparation.

NAAS will assess whether your figures meet our technical requirements by comparing each figure against our figure specifications.

# Author comment: Author response to Decision Letter 1

**DOI:** 10.1371/journal.pone.0361478.r002

28 Sep 2026

PLOS ONE revised submission

Manuscript: Clinical outcomes after fully visualized transforaminal endoscopic spine surgery in patients with lumbar disc herniation stratified by paraspinal muscle degeneration: a retrospective cohort study

Revision date: 19 September 2026

Dear Academic Editor and Reviewers,

Thank you for your careful and constructive assessment. We have revised the manuscript and resubmission package comprehensively. The major changes include a fully specified MRI grading and adjudication workflow with weighted kappa, covariate-adjusted longitudinal models treating degeneration grade as a categorical factor, false-discovery-rate control, MCID and partial R-squared context, complete cohort-flow reporting, descriptive recurrence and reoperation data, a fully anonymized English-language dataset, and separate Supporting Information tables. Each point is addressed below. Page and line numbers may shift during final typesetting; therefore, stable section, table, figure, and supporting-file locations are provided.

Summary of major revisions

�MRI assessment: two blinded spine surgeons independently graded bilateral multifidus fatty infiltration on axial T2-weighted MRI at the responsible mid-disc level; exact agreement was 95.3% and linearly weighted kappa was 0.967.

�Statistical analysis: longitudinal mixed models now adjust for age, sex, BMI, symptom duration, and surgical level and treat grade as a five-level factor; FDR q values are reported for the six primary grade-related tests.

�Clinical interpretation: per-grade effects and four-grade spans are compared with published MCIDs, partial R-squared values are reported, and the conclusions are framed as modest prognostic associations rather than a validated risk-stratification tool.

�Cohort and events: 136 patients were screened, 2 were excluded for incomplete records, 5 were lost to follow-up, and 129 were analyzed; three recurrences and one reoperation are reported.

�Transparency and data: the public workbook is English-language, exact age has been removed rather than hidden, and supplementary analyses are supplied separately as Supporting Information.

Responses to the Journal Requirements

Journal requirement 1

Comment: Please ensure that the manuscript meets PLOS ONE style and file-naming requirements.

Response: We revised the manuscript package to follow PLOS ONE conventions. The clean manuscript, marked manuscript, Response to Reviewers, Supporting Information tables, anonymized S1 Data workbook, and individual figure files are provided as separate, clearly named files. The reference list has been standardized to PLOS Vancouver style, including the first six authors followed by et al. when applicable.

Location: Throughout the manuscript and resubmission package.

Journal requirement 2

Comment: Please provide a complete Data Availability Statement.

Response: The Data Availability Statement has been revised to read: ‘All data are in the manuscript and/or supporting information files.’ The de-identified analytic data and data dictionary are supplied as S1 Data.

Location: Data Availability section; S1 Data.

Journal requirement 3

Comment: Remove or anonymize all personal information, including the age column, and do not hide columns containing personal information.

Response: Exact age was removed completely from the released workbook rather than hidden. The workbook contains no names, contact details, dates, hospital identifiers, hidden rows, or hidden columns. A simple sequential record number is used only to distinguish observations. The entirely blank Modified MacNab field was also omitted.

Location: S1 Data workbook, README and Data Dictionary sheets.

Journal requirement 4

Comment: Provide an English-language version of the dataset.

Response: All sheet names, variable names, coding descriptions, README text, and data-dictionary entries are now in English.

Location: S1 Data workbook.

Journal requirement 5

Comment: Remove supplementary tables from the manuscript, upload them as Supporting Information, and list their legends after the references.

Response: Supplementary Tables S1-S4 are supplied in a separate Supporting Information file. The manuscript retains only the legends for S1 Tables and S1 Data after the reference list.

Location: Supporting Information legends after the References; separate Supporting_Information_Tables file.

Journal requirement 6

Comment: Evaluate any reviewer-recommended citations for relevance; citation is not mandatory unless specifically required by the editor.

Response: We evaluated the suggested literature and added the directly relevant lumbar-MRI reliability study by Battaglia et al. and the lumbar-surgery MCID study by Copay et al. We also added the validation study for the simplified Chinese ODI version 2.1a by Liu et al. Other citations were retained only when they directly supported the corresponding statement.

Location: Methods—MRI grading and Outcomes; Discussion—clinical interpretation; References.

Reviewer 1

Comment 1

Comment: The manuscript contains an unresolved interobserver-reliability placeholder. How many observers were used, and what was the interobserver agreement?

Response: The internal note was removed. Two spine surgeons with 5-10 years of experience independently graded all scans while blinded to postoperative outcomes. Six disagreements were resolved by joint review with an adjudication panel comprising a spine surgeon and a radiologist, each with more than 10 years of experience. Exact agreement was 123/129 (95.3%); linearly weighted kappa was 0.967 (bootstrap 95% CI, 0.937-0.989), and quadratically weighted kappa was 0.985 (95% CI, 0.971-0.995). Repeat readings were not performed; therefore, intra-rater reliability was not assessed and this is acknowledged as a limitation.

Location: Abstract; Materials and methods—Paraspinal muscle degeneration grading; Discussion—Limitations.

Comment 2

Comment: Did any reoperations or recurrences occur during the 2-year follow-up? If unavailable, this should be highlighted as a limitation.

Response: Three recurrences were documented during the 2-year follow-up (2.3%): one in grade 3 and two in grade 4. One patient underwent reoperation. These events are reported descriptively because the small number precluded reliable risk modeling. Complication data were incomplete and were not assumed to be zero.

Location: Results—Patient characteristics; Discussion—Limitations.

Comment 3

Comment: Grade 0 contains only 13 patients, which limits statistical power when it is used as the reference group.

Response: We added group-specific sample sizes to Table 2 and explicitly acknowledged that the small grade 0 subgroup limits statistical power and the stability of grade 0-referenced contrasts.

Location: Table 2; Discussion—Limitations.

Reviewer 2

Comment 1

Comment: The complete-follow-up requirement may introduce selection bias. Please disclose the excluded subjects and consider whether age and other confounders may systematically favor particular degeneration groups.

Response: We now report the complete cohort flow: 136 patients were screened, 2 were excluded because of incomplete records, 5 were lost to follow-up, and 129 were analyzed. The flowchart displays these numbers. We explain that complete-case inclusion may preferentially exclude patients with complications, persistent dysfunction, or lower follow-up engagement. Baseline information for excluded patients was insufficient for a reliable grade-stratified comparison. The primary mixed models now adjust for age, sex, BMI, symptom duration, and surgical level.

Location: Abstract; Materials and methods—Study design and participants and Statistical analysis; Fig 2; Results—Patient characteristics; Discussion—Limitations.

Comment 2

Comment: Provide clinical context for the small but statistically significant effects by citing MCID values for VAS and ODI.

Response: We added Copay et al. and compare the estimates with MCIDs of 1.2 points for back-pain VAS, 1.6 for leg-pain VAS, and 12.8 for ODI. A one-grade increase corresponded to 0.114, 0.074, and 1.001 points, respectively; even the estimated four-grade spans remained below these MCIDs. We also clarify that MCIDs quantify within-patient change and are not directly equivalent to between-patient prognostic effects.

Location: Results—Adjusted associations with 2-year outcomes; Discussion—Clinical importance; Reference 24.

Comment 3

Comment: Clarify the MRI graders, their qualifications, blinding, and inter- and intra-rater reliability.

Response: The Methods now specify two independent blinded spine surgeons, their experience, the adjudication procedure, the MRI sequence, the evaluated muscle and slice, and both weighted-kappa estimates with 95% confidence intervals. Intra-rater reliability was not assessed because repeat readings were not performed, and this limitation is stated.

Location: Materials and methods—Paraspinal muscle degeneration grading; Discussion—Limitations.

Comment 4

Comment: Report how many patients were excluded because follow-up was incomplete and describe clinically meaningful events, including recurrence, reoperation, complications, MacNab outcomes, and JOA scores.

Response: We added the screened, excluded, lost-to-follow-up, and analyzed counts. Three recurrences and one reoperation are reported. Modified MacNab scores, JOA scores, and complication data were not recorded with sufficient completeness for reliable analysis; therefore, no complication count was inferred and these omissions are clearly listed as limitations.

Location: Fig 2; Results—Patient characteristics and Outcomes; Discussion—Limitations.

Minor comment 1

Comment: The data-access date 05/01/2026 appears to be in the future and may be a typographical error.

Response: The date is correct. To remove numeric ambiguity, it is now written as ‘1 May 2026.’

Location: Materials and methods—Study design and participants.

Minor comment 2

Comment: Explain why the time-by-grade interaction for leg pain was significant while the adjusted 2-year association was not significant.

Response: After covariate adjustment and factor coding, the leg-pain time-by-grade interaction was nominally significant (P = 0.036), but the FDR-adjusted q value was 0.053. Descriptive differences were concentrated at 6 months, whereas the adjusted 2-year association was not significant (P = 0.194). We therefore interpret the pattern as early or intermediate separation followed by convergence rather than a persistent long-term grade effect.

Location: Results—Linear mixed-effects model findings; Discussion—Leg pain and ODI.

Reviewer 3 Main Comments

Main comment 1

Comment: Remove the internal note and report the number, qualifications, and blinding of the MRI raters, together with inter- and intra-rater reliability.

Response: The internal note was removed. Two blinded spine surgeons with 5-10 years of experience independently graded all MRIs, and six disagreements were adjudicated by a senior spine surgeon and senior radiologist. Exact agreement, linearly weighted kappa, quadratically weighted kappa, and their confidence intervals are reported. Repeat readings were not performed, so intra-rater reliability was unavailable and is acknowledged.

Location: Abstract; Materials and methods—Paraspinal muscle degeneration grading; Discussion—Limitations.

Main comment 2

Comment: The longitudinal mixed models were not adjusted despite significant age differences across grades. Please adjust for age and other prespecified covariates or describe the models as unadjusted.

Response: All primary longitudinal models now adjust for age, sex, BMI, symptom duration, and surgical level in addition to categorical time, categorical grade, and their interaction. The adjusted models are described in Methods and reported in Table 3; interpretation is centered on adjusted estimates.

Location: Materials and methods—Statistical analysis; Results—Linear mixed-effects model findings; Table 3.

Main comment 3

Comment: The linear grade assumption may be inappropriate. Analyze grade as a factor or threshold, state whether sensitivity analyses were prespecified, and address multiple testing.

Response: The primary longitudinal models now treat grade as a five-level categorical factor. Two-year factor models and the grades 0-2 versus 3-4 threshold models are retained as post hoc sensitivity analyses and are explicitly labeled exploratory. Their nominal P values were not multiplicity-adjusted. For the six primary grade and time-by-grade tests, Benjamini-Hochberg FDR q values are reported.

Location: Materials and methods—Statistical analysis; Results—Linear mixed-effects model findings; Table 3; S3 Table.

Main comment 4

Comment: Report the partial R-squared or change in R-squared for degeneration grade, add MCID context, and avoid calling grade a risk-stratification marker without predictive-performance evidence.

Response: Table 4 now reports partial R-squared values for grade of 0.032, 0.014, and 0.045 and changes in adjusted R-squared of 0.025, 0.006, and 0.038 for back pain, leg pain, and ODI, respectively. MCID comparisons were added. ‘Risk-stratification marker’ was replaced by ‘modest prognostic association/correlate,’ and we explicitly state that predictive performance, calibration, and external validation were not evaluated.

Location: Results—Adjusted associations with 2-year outcomes; Table 4; Discussion; Conclusions.

Main comment 5

Comment: Report screened, excluded, and lost-to-follow-up numbers and acknowledge incomplete recurrence, reoperation, complication, MacNab, and JOA information.

Response: The flowchart now reports 136 screened, 2 excluded for incomplete records, 5 lost to follow-up, and 129 analyzed. Three recurrences and one reoperation are described. Complications, Modified MacNab scores, JOA scores, and rehabilitation adherence were incompletely recorded and are explicitly identified as limitations; the conclusions were correspondingly softened.

Location: Fig 2; Results—Patient characteristics; Discussion—Limitations and Conclusions.

Reviewer 3 Minor Comments

Minor comment 1

Comment: Use an unambiguous data-access date and correct the missing space after the date.

Response: The text now reads ‘1 May 2026,’ with an unambiguous date format and correct spacing.

Location: Materials and methods—Study design and participants.

Minor comment 2

Comment: Specify the MRI sequence, the muscle assessed, the exact slice, whether one or both sides were graded, and how bilateral findings were combined.

Response: We now specify axial T2-weighted MRI, the multifidus, the midpoint of the responsible intervertebral disc, bilateral assessment, and assignment of one overall grade from the combined bilateral appearance.

Location: Materials and methods—Paraspinal muscle degeneration grading.

Minor comment 3

Comment: In addition to the original Goutallier article, cite a lumbar-MRI adaptation or validation and report reliability.

Response: We added Battaglia et al., which evaluates the reliability of the Goutallier classification for lumbar multifidus fatty degeneration on MRI, and we report our interobserver weighted-kappa estimates.

Location: Materials and methods—Paraspinal muscle degeneration grading; Reference 18.

Minor comment 4

Comment: Because improvement-score analyses are mathematically coupled to baseline-adjusted 2-year models, consider moving them to Supporting Information.

Response: The improvement-score models were moved to S4 Table and are labeled post hoc supportive analyses. The manuscript explains the arithmetic coupling and bases primary inference on the adjusted 2-year residual-outcome models.

Location: Materials and methods—Statistical analysis; Discussion; S4 Table.

Minor comment 5

Comment: Add grade-specific group sizes to Table 2 or the Fig 3 legend.

Response: The grade-specific sample sizes (13, 25, 33, 32, and 26 for grades 0-4) were added to the Table 2 column headings.

Location: Table 2.

Minor comment 6

Comment: Define the VAS range and ODI version/language, describe follow-up collection, and report the number of operating surgeons and consistency of surgical/postoperative protocols.

Response: Back- and leg-pain VAS are now defined from 0 to 10. ODI is specified as the validated simplified Chinese version 2.1a scored from 0 to 100. Outcomes were obtained from inpatient and outpatient records; when a scheduled outcome was incompletely documented, trained research staff administered the same questionnaire by telephone. All procedures were performed by the same spine surgeon using consistent operative and postoperative protocols.

Location: Materials and methods—Surgical procedure and postoperative care; Outcomes.

Closing

We appreciate the editor’s and reviewers’ detailed guidance. The revision is substantially more transparent regarding selection, measurement reliability, confounding, multiplicity, effect magnitude, missing safety information, data sharing, and the limits of prognostic interpretation. We hope that the revised manuscript now meets PLOS ONE’s publication criteria.

Sincerely,

Dr. Zhang, on behalf of all authors.

Attachment Submitted filename: Response letter.docx

# Peer review: Decision Letter 1

**DOI:** 10.1371/journal.pone.0361478.r003

**Authors:** Rajakumar Anbazhagan

13 Oct 2026

Clinical outcomes after fully visualized transforaminal endoscopic spine surgery in patients with lumbar disc herniation stratified by paraspinal muscle degeneration: a retrospective cohort study

PONE-D-26-34472R1

Dear Dr. Zhang,

We’re pleased to inform you that your manuscript has been judged scientifically suitable for publication and will be formally accepted for publication once it meets all outstanding technical requirements.

Within one week, you’ll receive an e-mail detailing the required amendments. When these have been addressed, you’ll receive a formal acceptance letter and your manuscript will be scheduled for publication.

An invoice will be generated when your article is formally accepted. Please note, if your institution has a publishing partnership with PLOS and your article meets the relevant criteria, all or part of your publication costs will be covered. Please make sure your user information is up-to-date by logging into Editorial Manager at Editorial Manager® and clicking the ‘Update My Information' link at the top of the page. For questions related to billing, please contact billing support.

If your institution or institutions have a press office, please notify them about your upcoming paper to help maximize its impact. If they’ll be preparing press materials, please inform our press team as soon as possible -- no later than 48 hours after receiving the formal acceptance. Your manuscript will remain under strict press embargo until 2 pm Eastern Time on the date of publication. For more information, please contact onepress@plos.org.

Kind regards,

Academic Editor

PLOS One

Additional Editor Comments (optional):

Reviewers' comments:

Reviewer's Responses to Questions

**Comments to the Author**

Reviewer #1: All comments have been addressed

Reviewer #2: All comments have been addressed

Reviewer #3: All comments have been addressed

**********

2. Is the manuscript technically sound, and do the data support the conclusions?

Reviewer #1: Yes

Reviewer #2: Yes

Reviewer #3: Yes

**********

3. Has the statistical analysis been performed appropriately and rigorously?

Reviewer #1: Yes

Reviewer #2: Yes

Reviewer #3: Yes

**********

4. Have the authors made all data underlying the findings in their manuscript fully available?

The PLOS Data policy

Reviewer #1: Yes

Reviewer #2: Yes

Reviewer #3: Yes

**********

5. Is the manuscript presented in an intelligible fashion and written in standard English?

Reviewer #1: Yes

Reviewer #2: Yes

Reviewer #3: Yes

**********

Reviewer #1: (No Response)

Reviewer #2: (No Response)

Reviewer #3: The authors have comprehensively and satisfactorily addressed the previous review. I verified the point-by-point responses against the clean manuscript, and each major concern is resolved: grading reliability is now reported, the longitudinal models are covariate-adjusted with grade as a categorical factor and FDR control, the effect sizes are benchmarked against MCIDs with partial R-squared, the cohort flow and safety events are reported, and the interpretation is appropriately scaled to a modest prognostic association rather than a risk-stratification tool. Only minor copy-editing remains, and it does not require another round of review.

Please correct the typographical errors that I could spot: “recordes” → “records,” “Multifidua” → “Multifidus,” “Bilaterel” → “Bilateral,” “mulfifidus” → “multifidus” (Table 2), and “all outcome” → “all outcomes”; add the missing space in “0.967(95%CI” → “0.967 (95% CI”; standardise the coefficient notation (“beta = 0.074” → “β = 0.074”); remove the unnecessary comma in the Table 4 title (“between multifidus degeneration grade, and 2-year…”); remove the stray closing quotation mark in the Data Availability statement; and capitalise “han S” as “Han S” in reference 36.

**********

what does this mean?). If published, this will include your full peer review and any attached files.

If you choose “no”, your identity will remain anonymous but your review may still be made public.

**Do you want your identity to be public for this peer review?**  For information about this choice, including consent withdrawal, please see our Privacy Policy

Reviewer #1: **Yes:**  Pavithra Krishnaswami

Reviewer #2: No

Reviewer #3: No

**********

# Editor report: Acceptance letter

**DOI:** 10.1371/journal.pone.0361478.r004

**Authors:** Rajakumar Anbazhagan

PONE-D-26-34472R1

PLOS One

Dear Dr. Zhang,

I'm pleased to inform you that your manuscript has been deemed suitable for publication in PLOS One. Congratulations! Your manuscript is now being handed over to our production team.

At this stage, our production department will prepare your paper for publication. This includes ensuring the following:

* All references, tables, and figures are properly cited

* All relevant supporting information is included in the manuscript submission,

* There are no issues that prevent the paper from being properly typeset

You will receive further instructions from the production team, including instructions on how to review your proof when it is ready. Please keep in mind that we are working through a large volume of accepted articles, so please give us a few days to review your paper and let you know the next and final steps.

Lastly, if your institution or institutions have a press office, please let them know about your upcoming paper now to help maximize its impact. If they'll be preparing press materials, please inform our press team within the next 48 hours. Your manuscript will remain under strict press embargo until 2 pm Eastern Time on the date of publication. For more information, please contact onepress@plos.org.

You will receive an invoice from PLOS for your publication fee after your manuscript has reached the completed accept phase. If you receive an email requesting payment before acceptance or for any other service, this may be a phishing scheme. Learn how to identify phishing emails and protect your accounts at https://explore.plos.org/phishing.

If we can help with anything else, please email us at customercare@plos.org.

Thank you for submitting your work to PLOS One and supporting open access.

Kind regards,

PLOS One Editorial Office Staff

on behalf of

Dr. Rajakumar Anbazhagan

Academic Editor

PLOS One
